# Digital health interventions for lipid management in atherosclerotic cardiovascular disease: a systematic review and meta-analysis of randomised controlled trials

**DOI:** 10.1016/j.eclinm.2026.103886

**Published:** 2026-04-10

**Authors:** Youri Schut, Danique G.B. Buhler, Marjolein Snaterse-Zuidam, Arjan Malekzadeh, G. Aernout Somsen, Fabrice M.A.C. Martens, Michiel M. Winter

**Affiliations:** a Department of Cardiology, Heart Centre, Amsterdam University Medical Centre, Amsterdam, the Netherlands; b Department of Cardiology, Cardiology Centres of the Netherlands, the Netherlands; c Medical Library, Amsterdam University Medical Centre, Location Academic Medical Centre, Amsterdam, the Netherlands

**Keywords:** Digital health, Atherosclerotic cardiovascular disease, Low-density lipoprotein cholesterol, Lipid management, Secondary prevention

## Abstract

**Background:**

Atherosclerotic cardiovascular disease (ASCVD) remains the leading cause of morbidity and mortality worldwide. Lipid management is a key component of secondary prevention, but the majority of patients fail to achieve low-density lipoprotein cholesterol (LDL-c) goals. Digital health may serve as an innovative and cost-effective approach to improve lipid control, but a systematic quantitative synthesis is lacking. This study aimed to evaluate the effect of digital health interventions on LDL-c reduction and lipid target attainment in patients with ASCVD.

**Methods:**

A systematic search was performed in Medline, Embase, CINAHL, and CENTRAL from inception to May 27, 2025. Randomised controlled trials (RCTs) evaluating the effect of digital health interventions in patients with ASCVD and reporting lipid outcomes were included. The primary outcomes were change in mean LDL-c and achievement of lipid control (LDL-c < 1.8 mmol/L). Pooled estimates were calculated using random-effects models. Heterogeneity was assessed using the I^2^ statistic, and publication bias was assessed by visual inspection of a funnel plot and Egger’s test. The Cochrane Risk of Bias 2 was used to evaluate risk of bias. The protocol was registered with PROSPERO (CRD42024575196).

**Findings:**

Of the 4997 records identified, 18 RCTs from 11 regions (n = 12,970) were included in the systematic review. Risk of bias was scored as high across all included trials. Overall, digital health was associated with greater reductions in LDL-c compared with usual care [mean difference (MD) = −0.10 mmol/L (95% CI −0.17 to −0.04); p = 0.003; I^2^ = 74%]. No evidence of publication bias was detected. Lipid control was also improved [risk ratio (RR) = 1.15 (95% CI 1.03–1.28); p = 0.02; I^2^ = 57%]. In subgroup analyses, multi-component interventions showed the greatest reduction in LDL-c [MD = −0.22 mmol/L (−0.38 to −0.07); I^2^ = 78%], compared to SMS-only interventions [MD = −0.03 mmol/L (−0.14 to 0.08); I^2^ = 70%], SMS combined with supportive material [MD = −0.05 mmol/L (−0.14 to 0.03); I^2^ = 36%], and smartphone applications [MD = −0.07 mmol/L (−0.21 to 0.06); I^2^ = 47%], although differences between delivery modes were not statistically significant (p = 0.21). Self-reported medication adherence was slightly improved in the digital health group compared with controls [RR = 1.08 (1.00–1.16); p = 0.04; I^2^ = 88%]. In addition, digital health was associated with a reduced risk of hospitalisation [RR = 0.64 (0.50–0.81); p < 0.001; I^2^ = 18%].

**Interpretation:**

Our findings suggest that digital health interventions can support LDL-c reduction and lipid control in patients with ASCVD. Multi-component interventions were most effective, particularly when combined with high-frequency delivery, bidirectional communication, and telemonitoring. High heterogeneity and high risk of bias were observed across studies, and LDL-c reduction was not a primary outcome in most trials, warranting careful interpretation. Nevertheless, digital health shows potential for integration in lipid management, and warrants future research to optimise effectiveness and support implementation in clinical practice.

**Funding:**

Innovative Health Initiative Joint Undertaking.

**Research in context**

**Evidence before this study**

We searched PubMed, Embase, CINAHL, and Cochrane databases for systematic reviews published before 27 May 2025, using terms for digital health, atherosclerotic cardiovascular disease (ASCVD), and lipids, without language restrictions. We also searched the PROSPERO database using the same criteria. Several existing reviews examined digital health for cardiovascular disease more broadly, particularly in hypertension, diabetes, and heart failure, but no prior meta-analysis focused specifically on lipid management in ASCVD. A recent scoping review highlighted the potential of digital health on the current and future practice in lipid care, but pooled quantitative estimates were not provided.

**Added value of this study**

To our knowledge, we present the first quantitative synthesis of randomised controlled trials (RCT) assessing digital health interventions as part of lipid management in patients with established ASCVD. We synthesized results from 18 RCTs from 11 regions, including 12,970 participants, and found that digital health interventions are effective in reducing LDL-c and improving lipid target attainment. Effects were greatest for multi-component approaches. Additional intervention characteristics associated with the highest potential to reduce LDL-c included high-frequency delivery, bidirectional communication, and telemonitoring. Improvements in medication adherence and hospitalization rates were also observed.

**Implications of all the available evidence**

Digital health interventions are feasible and scalable tools that can improve lipid management within secondary prevention strategies in an ASCVD population. Although the current effect of these interventions remains modest, they offer potential to address the persistent gap between the recommended lipid goals and real-world attainment. Moreover, even small LDL-c reductions may translate into clinically meaningful reductions in recurrent cardiovascular events at population levels. Policymakers and clinicians should therefore consider integrating digital health tools into secondary prevention care pathways. Future research should prioritise trials specifically designed to target lipid management using digital health, with focus on optimising delivery modes, evaluating long-term clinical outcomes, and assessing cost-effectiveness and implementation strategies in routine practice.

## Introduction

Atherosclerotic cardiovascular disease (ASCVD), including stroke and coronary artery disease, remains the leading cause of death worldwide. Elevated low-density lipoprotein cholesterol (LDL-c) is a well-established modifiable risk factor for ASCVD,^1^^,^^2^ and intensive LDL-c lowering strongly reduces the risk of major adverse cardiovascular events (MACE).^3^^,^^4^ Accordingly, the 2019 European Society of Cardiology guidelines recommend an LDL-c reduction of at least 50% from baseline and a target value of <1.4 mmol/L (<55 mg/dL) for patients at very high cardiovascular (CV) risk.^5^ Despite the availability of effective lipid-lowering therapies and prevention strategies, real-world data indicate that many high-risk individuals fail to meet these lipid goals.^6^, ^7^, ^8^, ^9^ Therefore, a substantial number of patients unnecessarily remain at increased risk for recurrent CV events.

Given the rising burden of ASCVD,^10^ escalating healthcare costs, and growing shortages in healthcare personnel, there is urgent need for innovative, scalable approaches aimed at transforming secondary prevention strategies, particularly in lipid management. Digital health encompasses the use of information and telecommunication technologies to enhance patient care by improving access to medical data and delivering feedback to both patients and healthcare providers.^11^ Recent evidence suggests that digital health may support CV risk reduction across a broad range of cardiovascular disease.^12^, ^13^, ^14^ Despite these promising results, their application in lipid management remains underutilised and underexplored, with few trials designed to specifically target lipid outcomes.

A previous review provided insights on the impact of digital health on the current and future practice of lipid management,^15^ but was limited by broad inclusion criteria and the lack of quantitative syntheses. To date, a systematic synthesis of literature focused on digital health interventions for lipid management in ASCVD has not been published. Therefore, the aim of this review and meta-analysis was to evaluate the effect of digital health interventions on lipid management in patients with ASCVD, and to synthesise current evidence to inform future research and guide implementation into clinical practice.

## Methods

### Search strategy and selection criteria

This systematic review and meta-analysis were conducted in accordance with the 2020 Preferred Reporting Items for Systematic Reviews and Meta-Analyses (PRISMA) reporting guidelines.^16^ The protocol was prospectively registered in PROSPERO (CRD42024575196). In the registered protocol, the term ‘telemedicine’ was mainly used, although the operational definition corresponds to digital health interventions. The term ‘digital health’ is therefore used throughout this manuscript.

The objective was to evaluate the effectiveness of digital health interventions compared with usual care in patients with established ASCVD, with respect to lipid outcomes, medication adherence, and CV related outcomes, and to identify gaps in the current literature to inform future research and implementation. A medical information specialist (AM) systematically searched Medline, CINAHL, Embase, and Cochrane from inception to May 27, 2025. Search strategies included keywords and MeSH terms. The complete search strategy is provided in the Appendix (pp 2–9). Duplicate articles were removed by an in-house made deduplication tool (http://dedupendnote.nl:9777/). References were imported into the Rayyan web tool for removal of the remaining duplicates and screening of titles and abstracts, followed by full text screening for eligibility, all done independently by two authors (YS and DB). Discrepancies were resolved by consultation of a third author (MW).

Inclusion criteria were as follows: (1) adults (≥18 years old) with established ASCVD (defined as coronary heart disease (CHD), acute coronary syndrome (ACS), cerebrovascular disease, or peripheral artery disease (PAD)); and (2) the intervention consisted of digital health (e.g., text messaging, mobile applications, telemonitoring platforms, wearable-enabled monitoring, or web-based interventions) with a duration of at least 3 months; and (3) the study reported data on LDL-c and other lipid outcomes, regardless of whether LDL-c was the primary or secondary outcome; and (4) the comparator was usual care without any form of digital health; and (5) the study design was a randomised controlled trial (RCT) or observational cohort study. We excluded trials if no clear digital health intervention was described, the intervention comprised solely of telephone calls, and/or targeted a primary prevention population. Primary prevention populations were excluded since lipid targets, cardiovascular risk, and treatment intensity differ substantially compared to secondary prevention, which would introduce clinical heterogeneity and limit interpretability of the pooled estimates. A full list of inclusion and exclusion criteria is provided in the Appendix (p 10).

### Data analysis

Two authors (YS and DB) used a standardized template to extract the following data: country, study design, participant characteristics, follow-up duration, primary and secondary outcomes, and key features of the digital health intervention (delivery mode, frequency, communication, telemonitoring, acceptability and satisfaction). Corresponding study authors were contacted when additional information or clarification was required.

Digital health interventions were categorised based on their predominant mode of delivery as SMS-based interventions, SMS-based with supportive material, smartphone applications, or multi-component digital interventions. Multi-component interventions were defined as interventions integrating and combining multiple interactive care components.

The primary outcomes were (i) change in mean LDL-c (and 95% CI) at the longest available follow-up, and (ii) LDL-c target attainment (proportion achieving LDL-c threshold < 1.8 mmol/L). This threshold was applied uniformly to ensure comparability and consistency. The secondary outcomes included changes in high-density lipoprotein cholesterol (HDL-c), total cholesterol, triglycerides, medication adherence, and adverse events (CV-related events and hospital readmissions). All outcomes and analyses were prespecified in the protocol.

The quality of the studies was assessed independently by two authors (YS and DB) using the Cochrane risk-of-bias 2 tool,^17^ assessing the randomisation process, deviations from intended interventions, missing data, outcome measurement, and selective reporting.

### Statistical analysis

When trials reported outcomes at different follow-up time points, data from the longest available follow-up were used for analysis. All lipid outcomes were pooled across follow-up intervals ranging from 6 to 12 months. Heterogeneity across studies was explored quantitatively by using the I^2^ statistic and Cochran’s Q test. I^2^ values are categorized as low (I^2^ < 25%), moderate (I^2^ = 25%–50%), or high (I^2^ ≥ 50%) heterogeneity. Random-effects models were used for all outcomes regardless of the magnitude of heterogeneity across the studies. Effect sizes for continuous outcomes were expressed as mean difference (MD) with 95% confidence intervals (CI) and risk ratio (RR) with 95% CI for dichotomous outcomes. Forest plots were made to visualize the meta-estimates and pooled effects. Subgroup analyses were carried out based on delivery mode, delivery frequency (high: ≥4 contacts/week vs medium: <4 contacts/week), communication type (bidirectional vs unidirectional), presence of telemonitoring, follow-up duration (6 months vs > 6 months), baseline LDL-c levels (<2.6 mmol/L vs ≥ 2.6 mmol/L), and income economy, to explore potential sources of heterogeneity. Sensitivity analyses were done by applying fixed-effects models to assess the robustness of the findings. Publication bias was assessed by visual inspection of a funnel plot and Egger’s regression test for asymmetry.^18^ Statistical significance was defined as a two-sided p-value < 0.05. Data were analysed using ReviewManager version 5.4 and RStudio 4.3.2. Details on statistical conversions are provided in the Appendix (p 11).

### Ethics statement

As a systematic review and meta-analysis of previous published literature, this study did not involve primary data collection, and therefore did not require ethical approval and informed consent. All included trials reported institutional ethical approval in the original publications.

### Role of the funding source

This study received no dedicated funding. YS is supported by the Innovative Health Initiative (IHI) Joint Undertaking (grant number 101194785). The funder had no role in study design, data collection, data analysis, data interpretation, or writing of the report. All authors had full access to the data in the study and accept responsibility for the decision to submit for publication.

## Results

**Fig. 1:**
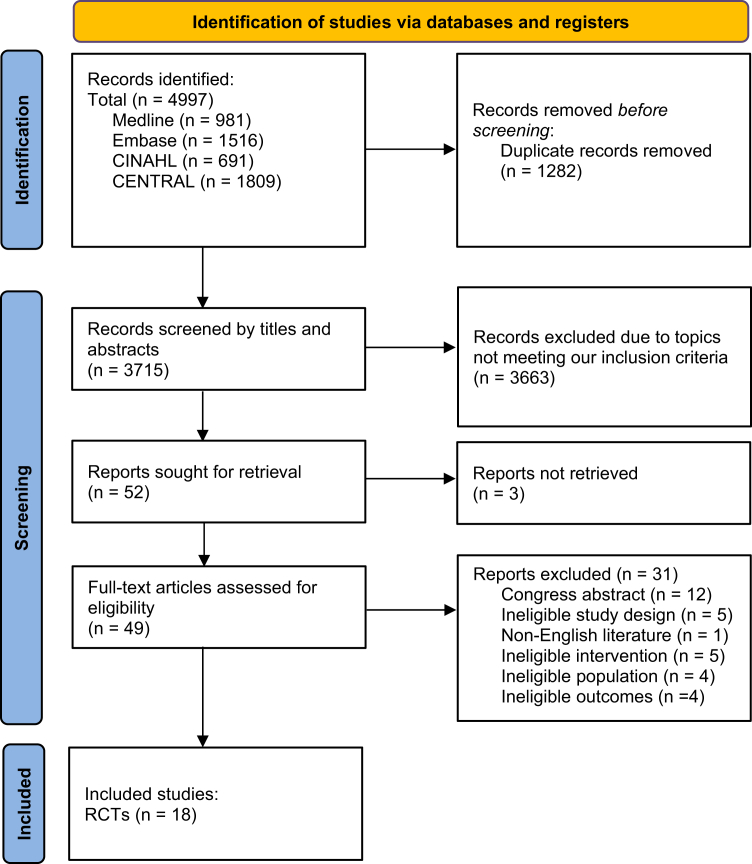
Prisma flow diagram of study selection.

The database search retrieved a total of 4997 articles that were screened for potential inclusion. We removed 1282 duplicates after which title and abstract screening was performed on 3715 articles. Full text screening for eligibility assessment was done on 49 articles. 31 full-text articles were excluded, and reasons for exclusion are listed in the Appendix (pp 12–14). Finally, 18 RCTs, published between 2012 and 2024, met the inclusion criteria and were included in the systematic review (Fig. 1).

### Characteristics of included studies

The main characteristics of the included studies are presented in Table 1. Sample sizes of the studies varied from 67 to 4298 participants and the total number of participants was 12,970. The mean age varied from 52.2 to 71.1 years and 24.7% (n = 3202) was female. Four trials^23^^,^^25^^,^^28^^,^^31^ included patients diagnosed with ACS, and nine trials^21^^,^^24^^,^^26^^,^^27^^,^^29^^,^^30^^,^^32^^,^^35^^,^^36^ included participants with CHD Participants with cerebrovascular disease were included in three trials,^19^^,^^20^^,^^34^ and two^22^^,^^33^ with a combination of clinical manifestations of atherosclerosis/arterial occlusive events. The trials were conducted in Australia,^23^^,^^24^^,^^27^ China,^26^^,^^29^^,^^30^^,^^34^^,^^36^ India,^19^^,^^20^ Hong Kong,^35^ New Zealand,^32^ South-Korea,^21^ South-America,^22^^,^^31^ and Europe.^25^^,^^28^^,^^33^ Nine studies^21^^,^^23^, ^24^, ^25^^,^^27^^,^^28^^,^^32^^,^^33^^,^^35^ were performed in high-income countries, seven^22^^,^^26^^,^^29^, ^30^, ^31^^,^^34^^,^^36^ in upper-middle-income countries, and two^19^^,^^20^ in lower-middle-income countries, as per World Bank classification (Appendix p 15).

**Table 1:** Characteristics and key findings of the included studies.

Author; country	Study design; follow-up duration	Study population	Patient characteristics^a^	Digital health intervention	Key findings
SPRINT INDIA (2023),^19^ India	RCT; 12 months	Ischaemic stroke or intracerebral haemorrhage	Intervention (n = 2148)/Control (n = 2150) Age: 56 (18–88)/56 (18–89) Female: 28.2%/26.6% LDL-c: 2.58 (1.06)/2.6 (1.08)	Regular SMS messages and videos promoting risk factor control and medication adherence, educational workbook	No between-group differences in LDL-c levels (p = 0.789) or other lipid values. Improvement in lifestyle behaviours, including alcohol and smoking cessation, and improvements in medication adherence. No reduction in vascular events.
Babu et al. (2024),^20^ India	RCT; 6 months	Recent stroke	Intervention (n = 105)/Control (n = 104) Age: 60.3 (11.5)/60.5 (10.2) Female: 23.8%/23.1% LDL-c: 2.6 (1.25)/2.45 (1.05)	Smartphone app with notifications and reminders on medication adherence, stroke awareness, lifestyle, and behavioural modifications	No between group differences in LDL-c levels (p = 0.297) or other vascular risk factors. Medication adherence and behavioural factors improved in digital health group.
Bae et al. (2021),^21^ South Korea	RCT; 6 months	CHD or post-PCI	Intervention (n = 440)/Control (n = 439) Age: 60.1 (10.6)/60.7 (10.4) Female: 16.4%/17.1% LDL-c: 2.87 (1.07)/2.84 (0.99)	Access to supporting website and SMS text messages regarding lifestyle modification, general cardiovascular health, and medication adherence	Medication adherence, physical activity, and healthy diet improved significantly in the digital health group. LDL-c values did not differ between groups (p = 0.12).
Bermon et al. (2021),^22^ Colombia	RCT; 12 months	ACS, stable angina, ischaemic cerebrovascular disease, PAD, and/or coronary revascularization	Intervention (n = 462)/Control (n = 468) Age: 64.0 (9.7)/63.1 (10.0) Female: 23.6%/19.7% LDL-c: 2.29 (0.98)/2.28 (0.97)	SMS text messaging focusing on health implications of adherence to healthy lifestyle and adequate use of prescribed medication	No significant change in LDL-c values at 12 months (p = 0.42). No impact observed on other CV risk factors.
Chow et al. (2022),^23^ Australia	RCT; 12 months	ACS	Intervention (n = 716)/Control (n = 708) Age: 58.0 (10.4)/58.0 (10.9) Female: 20.5%/21% LDL-c: 2.7 (1.06)/2.7 (1.12)	SMS text messaging with a customized and personalized program based on diet, physical activity capacity, and types of used medications	No effect on self-reported medication adherence (p = 0.15) or lipid outcomes. Small effects on lifestyle risk factors. No differences in CV events (p = 0.873)
Chow et al. (2015),^24^ Australia	RCT; 6 months	CHD	Intervention (n = 352)/Control (n = 358) Age: 57.9 (9.1)/57.3 (9.3) Female: 18.5%/17.6% LDL-c: 2.69 (1.01)/2.61 (0.91)	SMS text messaging with a lifestyle-focused semipersonalized support program on cardiovascular risk factors	Modest LDL-c reduction (p = 0.046) and improvements in other CV risk factors (p < 0.001). No improvement in medication adherence was observed.
Dalli et al. (2022),^25^ Spain	RCT; 10 months	ACS	Intervention (n = 33)/Control (n = 34) Age: 57.5 (9.0)/54.7 (9.9) Female: 12.9%/3.6% LDL-c: 1.53 (0.7)/1.4 (0.43)	Smartphone application for exercise planning and medication adherence and a webpage allowing personalized healthcare and tracking of patients adherence to guideline recommendations	No effects were observed in LDL-c (p = 0.8) or other lipid parameters. Improvements observed in physical activity.
Dorje et al. (2019),^26^ China	RCT; 12 months	CHD or post-PCI	Intervention (n = 156)/Control (n = 156) Age: 59.1 (9.4)/61.9 (8.7) Female: 18%/19% LDL-c: 2.0 (1.0)/1.91 (0.8)	Smartphone application in which patients received smartphone-based CR and secondary prevention programs delivered via social media	Significant improvements in LDL-c (p = 0.016), adherence to cardioprotective medications, blood pressure values, functional capacity and disease knowledge and awareness.
Gallagher et al. (2023),^27^ Australia	RCT; 6 months	CHD	Intervention (n = 194)/Control (n = 196) Age: 60.9 (11.9)/61.5 (11.2) Female: 18.6%/16.5% LDL-c: 2.4 (1.1)/2.2 (1.0)	Game-based mobile application to engage secondary prevention lifestyle behaviour and risk factor modification	Physical activity increased and triglycerides were lower in the digital health group (p = 0.004). No between-group differences in LDL-c, total cholesterol, and hospital admissions.
Krzowski et al. (2023),^28^ Poland	RCT; 6 months	Acute MI	Intervention (n = 50)/Control (n = 50) Age: 56.8 (9.23)/63.4 (11.4) Female: 32%/39% LDL-c: 3.04 (1.77)/2.89 (1.59)	Mobile application and digital system providing information on healthy lifestyle and CV risk factors, and short educational messages	LDL-c was lower in the digital health group, but not statistically significant (p = 0.323). No significant reduction in rehospitalization or other CV risk factors.
Li et al. (2022),^29^ China	RCT; 12 months	CHD	Intervention (n = 148)/Control (n = 152) Age: 61.4 (8.9)/62.3 (9.9) Female: 20.3%/24.5% LDL-c: 2.41 (0.88)/2.53 (0.85)	Self-management mobile application providing patient education, alerts for medical regimens, and self-recording of symptoms	LDL-c was significantly lower in the digital health group (p = 0.001). Medication adherence and the proportion of patients with controlled lipid and blood pressure values improved.
Lu et al. (2024),^30^ China	RCT; 12 months	CHD	Intervention (n = 629)/Control (n = 629) Age: 73.7 (10.4)/73.3 (9.7) Female: 36.5%/39.6% LDL-c: 2.75 (2.08–3.45)/2.69 (2.3–3.42)	A multi-parameter telemonitoring intervention using wearable devices, enabling remote physiological monitoring and virtual clinical management through a centralized telemedicine platform	LDL-c was significantly lower in the digital health group (p < 0.001). Additional CV risk factors improved as well. Lower occurrence in hospitalization and major adverse cardiovascular events (p < 0.001 for both).
Passaglia et al. (2022),^31^ Brazil	RCT; 6 months	ACS	Intervention (n = 90)/Control (n = 90) Age: 57.1 (9.3)/58 (10.5) Female: 27.8%/23.3% LDL-c: 2.68 (1.15)/2.48 (1.01)	SMS text messaging with advice on lifestyle, risk factor control, and medication adherence	No significant improvement in LDL-c, CV risk factor control, medication adherence, and adverse events.
Pfaeffli et al. (2015),^32^ New Zealand	RCT; 6 months	CHD	Intervention (n = 61)/Control (n = 62) Age: 59.0 (10.5)/59.9 (11.8) Female: 21%/16% LDL-c: 2.7 (1.3)/2.4 (1.0)	SMS text messaging with a supporting website delivering a comprehensive CR program, including patient education on CV risk factors and healthy lifestyle	A modest non-significant improvement in LDL-c (p = 0.053). Medication adherence strongly improved in the digital health group (p = 0.004). No significant effects on other lipid outcomes.
Vernooij et al. (2012),^33^ The Netherlands	RCT; 12 months	Clinical manifestation of atherosclerosis	Intervention (n = 164)/Control (n = 166) Age: 60.7 (7.8)/59.2 (8.9) Female: 22%/29% LDL-c 2 .8 (0.9)/2.8 (0.9)	Internet based, nurse-led risk factor management program with personalised self-management support, disease monitoring, and drug treatment guidance	Small effects on lowering vascular risk, but large reduction in LDL-c (p = 0.001). More patients quit smoking in the digital health group (p = 0.038).
Wang et al. (2020),^34^ China	RCT; 12 months	Stroke	Intervention (n = 100)/Control (n = 100) Age: 54.5 (7.9)^b^/54.6 (7.7)^b^ Female: 42%/37% LDL-c: 3.79 (1.52)/3.69 (1.43)	Brain and heart health manager-led mHealth follow-up, including patient application, telemonitoring of blood pressure and blood glucose, step count and exercise tracking	No significant effect on LDL-c (p = 0.455) or BMI. Blood pressure management and self-care ability improved.
Wong et al. (2020),^35^ China	RCT; 6 months	CHD	Intervention (n = 219)/Control (n = 219) Age: 52.2 (5.1)/52.5 (4.7) Female: 32.4%/36.6% LDL-c: 2.64 (0.56)/2.61 (0.56)	Interactive web based platform providing education on CV risk factors, personalized exercise plan, self-monitoring of health metrics, and features for progress tracking and alert system	Favourable effects on HDL-c and amount of exercise. No between-group differences in LDL-c (p = 0.40), blood pressure, triglycerides, and total cholesterol values.
Zheng et al. (2019),^36^ China	RCT; 6 months	CHD	Intervention (n = 411)/Control (n = 411) Age: 56.3 (9.3)/56.6 (9.7) Female: 14.1%/14.1% LDL-c: 2.44 (0.73)/2.56 (0.81)	Weekly educational and motivational SMS text messaging on disease knowledge, CV risk factors, medication adherence, and physical activity	Non-significant trend toward lower LDL-c (p = 0.056). No significant effects on other CV risk factors.

ACS, acute coronary syndrome; BMI, body-mass index; CHD, coronary heart disease; CR, cardiac rehabilitation; CV, cardiovascular; HDL-c, high-density lipoprotein cholesterol; LDL-c, low-density lipoprotein cholesterol; mHealth, mobile health; MI, myocardial infarction; PAD, peripheral artery disease; PCI, percutaneous coronary intervention; RCT, randomised controlled trial; SMS, short message service.

a Age (years) and LDL-c (mmol/L) are reported as mean with standard deviation (SD) or median (25th percentile–75th percentile).

b Age was reported in categories (40–49, 50–59, and 60–69 years); mean age was estimated from grouped data.

### Assessment of bias of the included articles

All 18 included articles were categorised as high risk, predominantly due to performance bias, as blinding of participants and personnel was not feasible given the nature of digital health interventions. Random sequence generation was adequately performed in all studies and therefore risk of selection bias was classified as low. Allocation concealment and blinding of outcome assessment were scored as low risk in most trials, although one study^32^ did not blind their outcome assessors, and four studies^27^^,^^29^^,^^33^^,^^34^ did not provide complete information. Attrition rates were generally comparable between groups, and incomplete outcome data were handled by intention-to-treat analyses in most studies, though two trials^28^^,^^31^ were scored as high risk for attrition bias (Appendix pp 16–17).

### Description of digital health interventions

An extensive description of the digital health interventions is provided in Table 2. Large heterogeneity was observed between the different interventions, mainly consisting of 4 categories: SMS text messaging only,^22^, ^23^, ^24^^,^^31^^,^^36^ SMS text messaging with supportive material,^19^^,^^21^^,^^32^ smartphone applications,^20^^,^^25^^,^^27^, ^28^, ^29^ and multi-component platforms.^26^^,^^30^^,^^33^, ^34^, ^35^ Nine trials^20^^,^^25^^,^^26^^,^^28^, ^29^, ^30^^,^^32^, ^33^, ^34^ used telemonitoring as a component of the intervention, mainly focusing on monitoring blood pressure, heart rate, glucose, and body weight. The frequency of delivery was classified as either high- or medium-frequency, and type of communication as unidirectional or bidirectional. Seven trials^19^^,^^21^^,^^23^^,^^24^^,^^26^^,^^32^^,^^36^ documented utility and perceived acceptability of the digital health intervention. Follow-up duration differed substantially in the trials, ranging from 6 to 12 months. Half of the included trials^20^^,^^21^^,^^24^^,^^27^^,^^28^^,^^31^^,^^32^^,^^35^^,^^36^ had a short-term follow-up duration (6 months), and the other half^19^^,^^22^^,^^23^^,^^25^^,^^26^^,^^29^^,^^30^^,^^33^^,^^34^ had a long-term follow-up duration (>6–12 months).

**Table 2:** Delivery characteristics and user acceptability of the included digital health interventions.

Study	Digital health category	Duration	Frequency	Communication	Telemonitoring	Key features	Utility and perceived acceptability
SPRINT INDIA et al. (2023)^19^	SMS intervention with supportive material	12 months	Medium	Unidirectional	N/A	Unidirectional SMS with short videos and educational workbook	- SMS messages viewed at 6 weeks: 70.2%, at 1 year: 36% - Video messages viewed at 6 weeks: 54.2%, at 1 year: 32.4% - Conclusion: Emphasizing feasibility, although adherence strongly decreases over 1-year
Babu et al. (2024)^20^	Smartphone app	6 months	Medium	Bidirectional; patients receive personalized feedback based on personal vascular risk factors	Yes; patients uploading vascular risk factor outcomes to the app	Smartphone app with educational materials on secondary stroke prevention, medication reminders, and lifestyle guidance. SMS messaging for education, reminders, and motivational support	N/A
Bae et al. (2021)^21^	SMS intervention with supportive material	6 months	High	Unidirectional; automated program without interaction between patient and provider	N/A	Semipersonalized SMS text messages with additional web platform (use of this platform was optional)	- Messages helpful: 82%. - Messages easy to understand: 94.6%. - Motivated lifestyle change: 78.2%. - Conclusion: Overall, satisfaction with frequency, timing and duration of program; effective for lifestyle motivation
Bermon et al. (2021)^22^	SMS intervention only	12 months	Medium	Unidirectional; no reply mechanism	N/A	SMS text messaging without supportive websites and/or apps	N/A
Chow et al. (2015)^24^	SMS intervention only	6 months	High	Unidirectional	N/A	SMS text messaging without supportive websites and/or apps	- Useful: 91%. - Easy to understand: 97%. - Motivated to change diet: 81%. - Motivated to improve physical activity: 73%. - Conclusion: Highly effective and feasible; strong motivation for diet and physical activity changes
Chow et al. (2022)^23^	SMS intervention only	12 months	Medium	Bidirectional; participants were able to reply to messages, responses were reviewed and answered	N/A	Personalized SMS text messaging on lifestyle, medication, and secondary prevention, without supportive websites and/or apps	- Useful: 86%. - Easy to understand: 94%. - Motivation to change lifestyle and reminder to take medication: 63%. - Conclusion: Consistent satisfaction; moderate motivation for medication adherence and lifestyle change
Dalli Peydro et al. (2022)^25^	Smartphone app	10 months	High	Bidirectional; healthcare team monitored incoming data on web platform, patients were contacted if threshold for risk factors were exceeded	Yes; heart rate monitor for exercise intensity tracking	Smartphone app for medication adherence and lifestyle modification, and web platform with educational elements, adherence tracking, and personalized healthcare	N/A
Dorje et al. (2019)^26^	Multi-component	6 months	High	Bidirectional; feedback loops by clinicians/coached based on personal data	Yes; continuous monitoring of blood pressure, heart rate, and activity by step count, with data automatically transported to portal	Smartphone-based programme via WeChat patient-provider platform with modules with educational modules, activity tracking	- Message/modules received: 100%. - >75% of modules/messages read: 95%. - Improved CR and secondary prevention understanding: 95%. - Improved dietary habits, adherence to physical activity, improved medication adherence: 97%. - Continue using program >6 months: 98% - Conclusion: Extremely high engagement, sustained use, and satisfaction; strong clinical and behavioural benefits
Gallagher et al. (2023)^27^	Smartphone app	6 months	High	Unidirectional; app provides automated prompts, feedback, and reminders, not clinician driven	N/A	Gamified smartphone application (MyHeartMate) for health tracking, includes challenges, reward mechanisms, and congratulatory messages	N/A
Krzowski et al. (2023)^28^	Smartphone app	6 months	High	Bidirectional; patients’ reported and incoming vital signs are analysed, following by feedback if necessary	Yes; monitoring of blood pressure, heart rate, physical activity and body weight via connected devices or manual input	Smartphone app (afterAMI) with vital signs reporting, medication reminders, educational content, and lifestyle advice	N/A
Li et al. (2022)^29^	Smartphone app	12 months	High	Bidirectional; incoming data is reviewed by physicians, medication and lifestyle plans are updated based on the personal data	Yes; automatic transfer of heart rate and blood pressure to patient portal	Smartphone app with self-management platform, medication taking reminders, symptom tracking	N/A
Lu et al. (2024)^30^	Multi-component	12 months	High	Bidirectional; physiological data was assessed and personalized feedback was provided such as medication adjustments and lifestyle advice	Yes; blood pressure, blood glucose, and electrocardiogram	Multimedia, app-based and wearable device intervention for tracking of vital parameters	N/A
Passaglia et al. (2022)^31^	SMS intervention only	6 months	High	Unidirectional; participant were not able to reply to messages, no communication with providers	N/A	SMS messages containing education and motivation on medication adherence, lifestyle	N/A
Pfaeffli et al. (2015)^32^	SMS intervention with supportive material	6 months	High	Bidirectional; personalized feedback/response based on step count data, option for participants to ask questions and feedback on behaviour	Yes; pedometer for step count	SMS with additional supporting website containing educational materials	- High engagement and utility; effective in improving disease knowledge and supporting recovery
Vernooij et al. (2012)^33^	Multi-component	12 months	Medium	Bidirectional; nurse practitioner reviewed incoming data and medication adjustments were done, adherence was tracked and replied to messages	Yes; multiple risk factors such as blood pressure, weight, smoking behaviour, cholesterol, and glucose were submitted	Personalized web-based online platform for vascular risk factor management, nurse messaging, and educational material	N/A
Wang et al. (2020)^34^	Multi-component	12 months	High	Bidirectional; personalized feedback and personalized interventions were provided by health managers based on analysed patient data	Yes; blood pressure, blood glucose, step count, and exercise tracking	Multiplatform intervention integrating smartphone app, telemonitoring by devices, and online support system for health education	N/A
Wong et al. (2020)^35^	Multi-component	6 months	Medium	Unidirectional; no real-time communication between participant and healthcare provider, but automated system response	N/A	Website-based platform for education, self-monitoring, and automated reminders	N/A
Zheng et al. (2019)^36^	SMS intervention only	6 months	High	Unidirectional; no real-time interactive communication or tracking of messages or personal data	N/A	SMS text messages containing information about disease knowledge, medication adherence, blood pressure control, and lifestyle	- Messages useful: 96.1% - Easy to understand: 98.8% - >75% of messages read: 80.3% - Willingness to continue: 94% - Conclusion: Very high user satisfaction and feasibility; strong potential for ongoing engagement

N/A, not applicable; SMS, short message service.

### LDL-c and lipid control

**Fig. 2:**
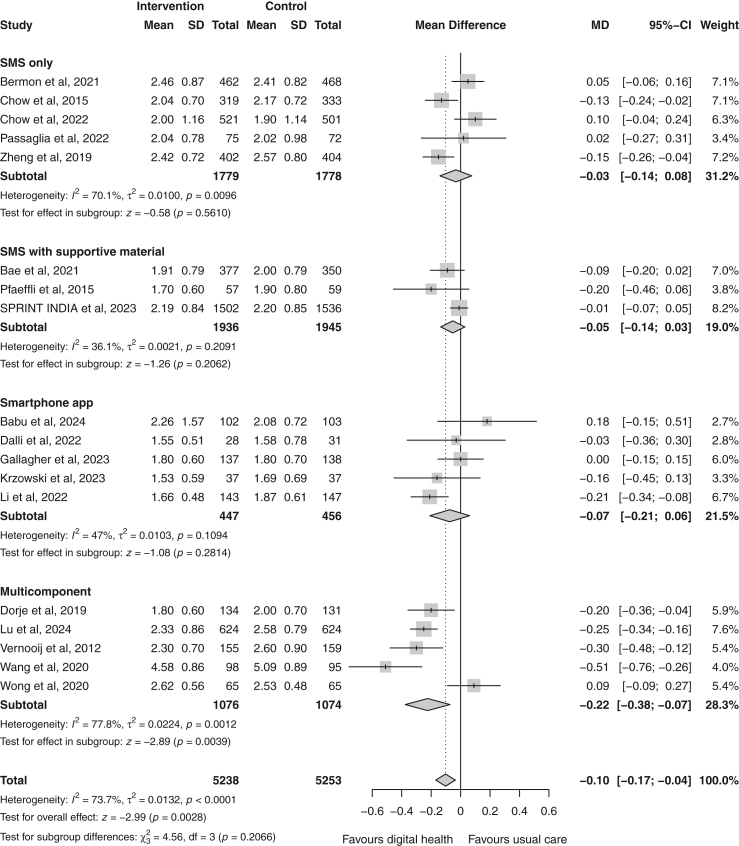
Forest plot of the mean difference in low-density lipoprotein cholesterol (mmol/L) between the digital health and usual care groups, stratified by delivery mode. Follow-up ranged from 6 to 12 months. Squares represent study-specific estimates and horizontal lines represent 95% confidence intervals. The diamonds represent the pooled effect estimate. CI, confidence interval; I^2^, measure of statistical heterogeneity; MD, mean difference; SMS, short message service.

**Fig. 3:**
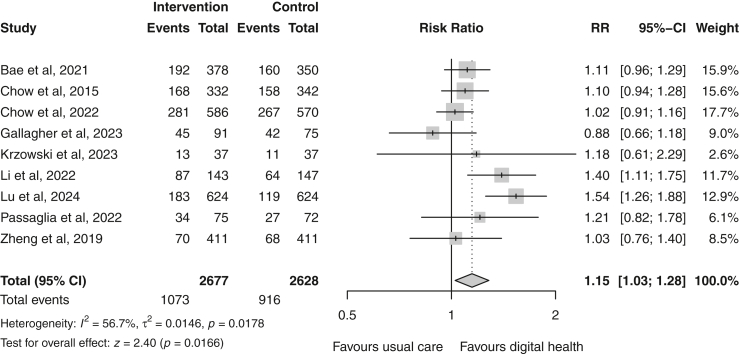
Forest plot of lipid target attainment (LDL-c < 1.8 mmol/L) in the digital health versus usual care groups. Squares represent study-specific estimates and horizontal lines represent 95% confidence intervals. The diamond represents the pooled effect estimate. CI, confidence interval; I^2^, measure of statistical heterogeneity; RR, risk ratio.

LDL-c changes between the two groups were reported in all 18 included trials involving 10,491 patients. Overall, the digital health intervention group had a −0.10 mmol/L (95% CI −0.17 to −0.04) significant greater reduction in LDL-c compared with the usual care group (Fig. 2). The pooled heterogeneity across studies was significant (χ^2^ = 65.5; p < 0.00001) and high in magnitude (I^2^ = 74%), indicating substantial variability between studies. Multi-component interventions revealed the greatest LDL-c reduction (MD = −0.22 mmol/L [−0.38 to −0.07]) compared to interventions that used smartphone apps (MD = −0.07 mmol/L [−0.21 to 0.06]), that employed SMS only (MD = −0.03 mmol/L [−0.14 to 0.08]), and SMS combined with supportive components (MD = −0.05 mmol/L [−0.14 to 0.03]) (Fig. 2). However, the test for subgroup differences was not statistically significant (p = 0.21). Nine studies^21^^,^^23^^,^^24^^,^^27^, ^28^, ^29^, ^30^, ^31^^,^^36^ reported outcomes regarding achieving the recommended target of LDL-c < 1.8 mmol/L. Participants in the digital health group were 1.15 times more likely to achieve this target compared to usual care (random-effects RR 1.15 [95% CI 1.03–1.28]; p = 0.02; I^2^ = 57%; n = 5305) (Fig. 3).

### Other lipid parameters

**Fig. 4:**
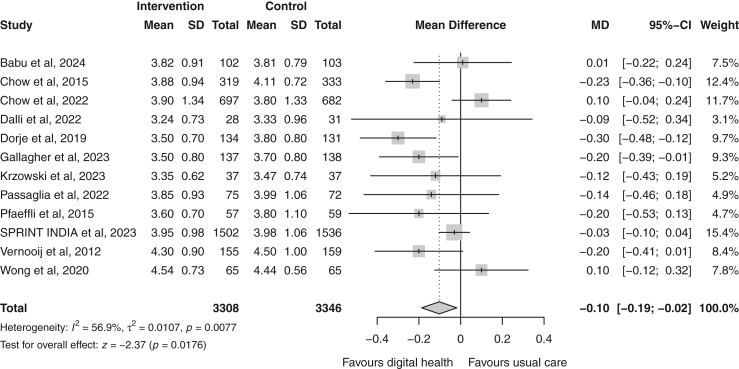
Forest plot of the mean difference in total cholesterol (mmol/L) between the digital health and usual care groups. Squares represent study-specific estimates and horizontal lines represent 95% confidence intervals. The diamond represents the pooled effect estimate. CI, confidence interval; I^2^, measure of statistical heterogeneity; MD, mean difference.

**Fig. 5:**
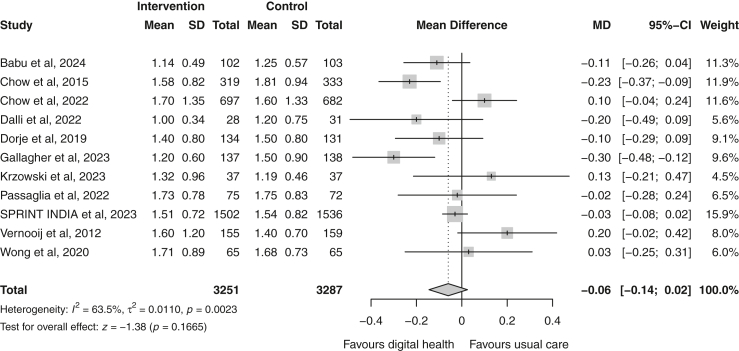
Forest plot of the mean difference in triglycerides levels (mmol/L) between the digital health and usual care groups. Squares represent study-specific estimates and horizontal lines represent 95% confidence intervals. The diamond represents the pooled effect estimate. CI, confidence interval; I^2^, measure of statistical heterogeneity; MD, mean difference.

**Fig. 6:**
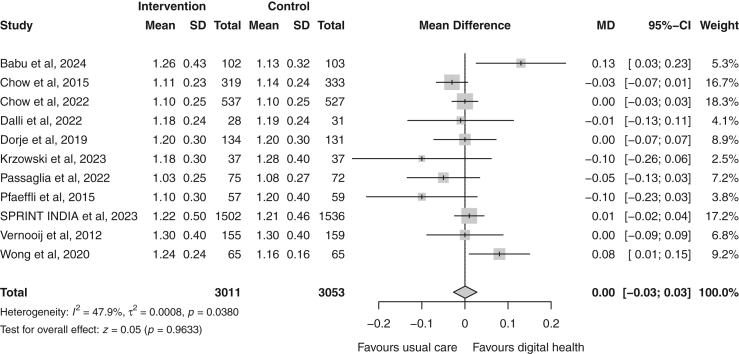
Forest plot of the mean difference in high-density lipoprotein cholesterol (mmol/L) between the digital health and usual care groups. Squares represent study-specific estimates and horizontal lines represent 95% confidence intervals. The diamond represents the pooled effect estimate. CI, confidence interval; I^2^, measure of heterogeneity; MD, mean difference.

Total cholesterol was reported by 12 trials.^19^^,^^20^^,^^23^, ^24^, ^25^, ^26^, ^27^, ^28^^,^^31^, ^32^, ^33^^,^^35^ A greater and statistically significant reduction in total cholesterol was observed in the digital health group compared to usual care (random effect MD −0.10 [−0.19 to −0.02] mmol/L; p = 0.02; I^2^ = 57%; n = 6654) (Fig. 4). Eleven trials reported changes in HDL-c^19^^,^^20^^,^^23^, ^24^, ^25^, ^26^^,^^28^^,^^31^, ^32^, ^33^^,^^35^ and triglycerides.^19^^,^^20^^,^^23^, ^24^, ^25^, ^26^, ^27^, ^28^^,^^31^^,^^33^^,^^35^ Meta-analyses demonstrated a modest, but non-significant reduction in triglyceride levels in the digital health group (random-effects MD −0.06 [−0.14 to 0.02] mmol/L; p = 0.17; I^2^ = 63%; n = 6538) (Fig. 5) and no change in HDL-c was observed (random-effects MD 0.00 [−0.03 to 0.03] mmol/L; p = 0.96; I^2^ = 48%; n = 6064) (Fig. 6).

### Adherence to medication

**Fig. 7:**
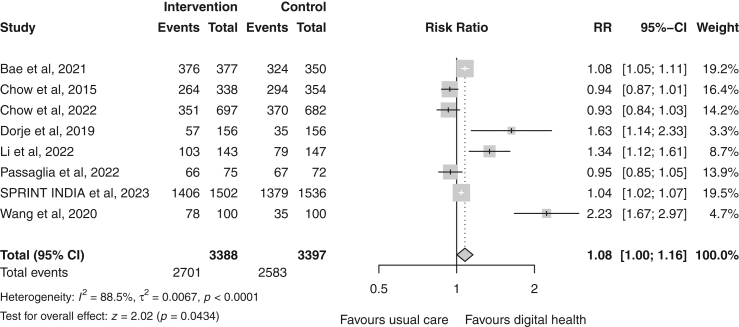
Forest plot of the dichotomous outcome measurements for medication adherence in the digital health versus usual care groups. Squares represent study-specific estimates and horizontal lines represent 95% confidence intervals. The diamond represents the pooled effect estimate. CI, confidence interval; I^2^, measure of statistical heterogeneity; RR, risk ratio.

A total of 11 studies^19^, ^20^, ^21^, ^22^, ^23^, ^24^^,^^26^^,^^29^^,^^31^^,^^32^^,^^34^ reported data on self-reported medication adherence of which eight were suitable for meta-analysis. Meta-analyses showed that the digital health group was 1.08 times more likely to achieve medication adherence, a modest but statistically significant effect compared with usual care (random-effects RR 1.08 (95% CI 1.00–1.16); p = 0.04; I^2^ = 88%; n = 6785) (Fig. 7). This finding should be interpreted with caution given the considerable heterogeneity across studies. One study^20^ used the 4-item Morisky Medication Adherence Scale (MMAS),^37^ and demonstrated a significant improvement in medication adherence in the digital health group compared to usual care (mean score 3.61 ± 0.95 vs 2.88 ± 1.32; p < 0.001). Another study^32^ used the 8-item MMAS, and demonstrated similar results (mean score 7.3 ± 0.9 vs 6.8 ± 1.2; p = 0.004).

### Adverse events

**Fig. 8:**
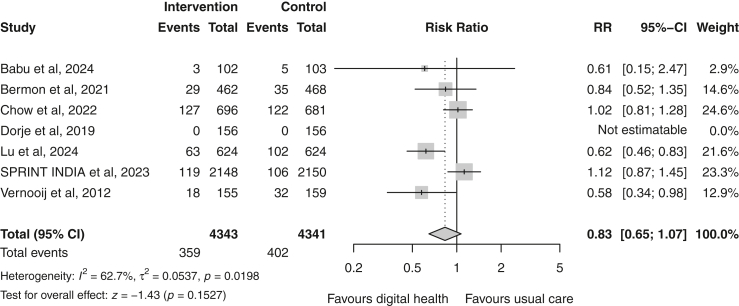
Forest plot of (composite) major adverse cardiovascular events in the digital health versus usual care groups. Squares represent study-specific estimates and horizontal lines represent 95% confidence intervals. The diamond represents the pooled effect estimate. CI, confidence interval; I^2^, measure of heterogeneity; RR, risk ratio.

**Fig. 9:**
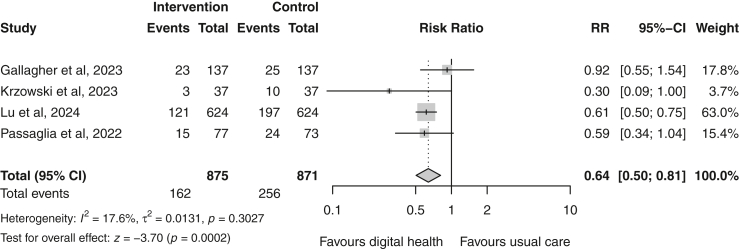
Forest plot of (re)hospitalisation events in the digital health versus usual care groups. Squares represent study-specific estimates and horizontal lines represent 95% confidence intervals. The diamond represents the pooled effect estimate. CI, confidence interval; I^2^, measure of heterogeneity; RR, risk ratio.

Seven trials^19^^,^^20^^,^^22^^,^^23^^,^^26^^,^^30^^,^^33^ reported data on (a composite of) CV events and four studies^27^^,^^28^^,^^30^^,^^31^ on (re)hospitalisation events. Digital health showed no significant effect on CV events in the pooled estimate (random-effects RR 0.83 [95% CI 0.65–1.07]; p = 0.15; I^2^ = 63%; n = 8684) (Fig. 8). In contrast, a significant reduction in the risk for (re)hospitalisation events was observed in the digital health group (random-effects RR 0.64 [95% CI 0.50–0.81]; p < 0.001; I^2^ = 18%; n = 1746) (Fig. 9).

### Subgroup and exploratory analyses

We explored study-level moderators to account for heterogeneity, including frequency of delivery, mode of communication, use of telemonitoring, duration of intervention, baseline LDL-c values, and income-economy to explore other potential sources of heterogeneity. Differences between subgroups were not statistically significant except for characteristics of the digital health interventions. LDL-c reductions were greater in studies incorporating telemonitoring (−0.22 mmol/L [−0.30 to −0.14]) compared to those without (−0.03 mmol/L [−0.08 to 0.03]) (Appendix p 18). Studies using bidirectional communication were also associated with greater reductions (−0.17 mmol/L [−0.28 to −0.06]) compared to unidirectional interventions (−0.04 mmol/L [−0.10 to 0.02]) (Appendix p 19). Finally, studies with high-frequency interventions were associated with greater LDL-c reductions (−0.16 mmol/L [−0.22 to −0.04]) compared with medium-frequency interventions (0.01 mmol/L [−0.09 to 0.11]) (Appendix p 20). Duration of intervention, income-economy, and baseline LDL-c did not explain heterogeneity, although long-term interventions, higher LDL-c values at baseline (≥2.6 mmol/L), and upper-middle income economies showed greatest LDL-c reductions (Appendix pp 21–23).

### Sensitivity analyses

The robustness of our findings were assessed by testing whether the fixed-effects model would yield different results from the random-effects model for LDL-c. The fixed-effects model produced a similar pooled estimate for LDL-c reduction (−0.09 mmol/L [−0.12 to −0.06]) with narrower confidence intervals, indicating that these results were not driven by model (Appendix p 24). Nonetheless, smartphone application interventions became statistically significant when applying a fixed-effects model (−0.10 mmol/L [−0.19 to −0.02], p = 0.02). Additionally, we removed each study sequentially to test whether the direction of the pooled estimate was driven by a single study. The pooled effect for LDL-c remained consistent, and statistical significance was not affected with the removal of an individual study.

### Publication bias

We visually explored publication bias using funnel plots for the LDL-c outcome. Visual assessment of the funnel plot for studies reporting LDL-c changes did not suggest publication bias (Appendix p 25), which was supported by Egger’s regression test for plot symmetry (p = 0.916).

## Discussion

The aim of this systematic review and meta-analysis was to provide a synthesis of the effectiveness of digital health interventions versus usual care for lipid management in patients with ASCVD. The meta-analysis of 18 RCTs (n = 12,970 participants) identified that digital health interventions resulted in greater LDL-c reductions than standard care. Although the absolute decrease in LDL-c was modest (−0.10 mmol/L), it was statistically robust and accompanied by a 15% higher likelihood of reaching target thresholds (<1.8 mmol/L). Given that the majority of trials were not focused on LDL-c reduction as primary outcome, this degree of LDL-c improvement is promising and can offer small but consistent additional benefits over usual care in secondary prevention.

The modest magnitude of LDL-c lowering warrants clinical interpretation, particularly in a very high-risk ASCVD population. Current guidelines recommend intensive lipid lowering, with LDL-c reductions of at least 50% from baseline and absolute targets below 1.4 mmol/L,^5^ and therefore the observed effect is small compared with pharmacological lipid-lowering therapies. Large meta-analyses^38^ have shown a 22% relative risk reduction in MACE per 1 mmol/L decrease in LDL-c, indicating that the estimated individual-level risk reduction associated with the observed effect is limited. Nevertheless, the effect was accompanied by a significant improvement in lipid target attainment. Notably, even small LDL-c reductions may translate into meaningful population-level benefits when scalable interventions are used broadly. Digital health interventions should therefore be considered as a complementary strategy to standard care, with potential to enhance therapy adherence, support lifestyle modification, improve access to care, and facilitate timely treatment optimisation.

Substantial heterogeneity was observed across trials (I^2^ = 74%), likely explained by variations in intervention designs, such as delivery mode, intensity, duration, communication type, use of remote patient monitoring, and whether LDL-c reduction was the primary goal. Pooling such diverse approaches may have attenuated the overall effect size. When comparing the different modalities, we found that multi-component interventions were more effective in terms of LDL-c reduction compared to interventions using only SMS and smartphone apps. In addition, higher-frequency delivery, bidirectional communication, and use of telemonitoring were also associated with larger LDL-c reductions. These findings are in line with a recent systematic review where phone text messaging for secondary prevention was not associated with a significant effect on LDL-c outcomes,^39^ highlighting the limited potential for lipid management. In contrast, multi-component interventions offer a great range of functionalities, such as automated feedback loops, interactive interfaces, and real-time tracking of health data, which are naturally lacking with interventions using SMS text messaging alone. Such integrated approaches typically combine effective components, such as smartphone apps, messaging, educational content, reminders, and wearables, often allowing the opportunity for bidirectional communication and telemonitoring. These findings highlight the differential effectiveness of various digital health modalities and characteristics, with more interactive, personalised, and continuous interventions having greater LDL-c lowering potential.

Digital health interventions should be considered as strategies rather than direct lipid-lowering interventions, supporting behaviours and care processes that ultimately influence lipid management. Therefore, the observed LDL-c reductions are likely mediated through indirect mechanisms, such as medication adherence and lifestyle change. A critical component of effective secondary prevention and lipid management is adherence to medical therapy.^6^^,^^40^, ^41^, ^42^ Sustained medication adherence has been associated with a 20% lower risk of cardiovascular disease, with an estimated 9% of CV events attributable to poor adherence.^43^ Previous systematic reviews have evaluated the effectiveness of digital health interventions on medication adherence, and reported substantial improvements in adherence to medication.^44^, ^45^, ^46^ In contrast, our meta-analysis showed a modest but significant improvement in adherence in the digital health group. The smaller effect size could be explained by reliance on self-reported measures and the unblinded design in most trials, both prone to bias. Notably, trials that used validated instruments such as the MMAS, demonstrated greater improvements in adherence.^20^^,^^32^ Beyond medication adherence, many interventions targeted lifestyle behaviour including physical activity and diet, which may have further contributed to LDL-c reductions. While lifestyle outcomes were not systematically assessed, increased engagement with lifestyle modification likely complemented pharmacological management, partially explaining the observed LDL-c effects.

Strengths of our study include the use and adherence of rigorous standard methodology as documented in the PRISMA and Cochrane guidelines. Next to that, our study population includes a large, demographically and culturally diverse population from 11 regions, representing lower-middle-income, upper-middle-income, and high-income economy countries from four continents. This geographic diversity strengthens the generalizability of our findings and highlights the potential of digital health in remote regions with limited access to healthcare resources. Furthermore, we performed a comprehensive series of subgroup and sensitivity analyses to ensure robustness of the overall effect size, and included data on the participants perceived acceptability and utility of the digital health interventions. Lastly, we reported not only the reduction in LDL-c, but also the proportion of participants achieving lipid control, defined by LDL-c < 1.8 mmol/L. Although this target is not in accordance with the current international guidelines, it nevertheless provides relevance to clinical decision-making and risk factor management.

Our study has several limitations. First, most studies were not specifically designed to target lipid management, therefore LDL-c was frequently being assessed as secondary instead of primary endpoint. This may increase imprecision and selective reporting despite LDL-c being objectively measured. Second, substantial heterogeneity was observed across pooled analyses, particularly for change in LDL-c, indicating variability in intervention design and follow-up duration. This heterogeneity limits certainty of the overall effect estimate and findings should be interpreted with caution. However, random-effects models and subgroup analyses were applied to account for this. Third, all studies lacked blinding of both patients and caregivers, introducing performance bias, potentially influencing lifestyle and medication-taking behaviour. Due to the nature of digital health interventions, blinding of participants and healthcare personnel is practically impossible. Moreover, some studies had small sample sizes, further limiting the reliability of the overall estimates. Fourth, classification of digital interventions was challenging due to overlap in modalities, potentially influencing the subgroup estimates. The authors aimed to mitigate this potential bias by classifying interventions according to their predominant delivery mode. Furthermore, no trials investigated interventions and follow-up durations beyond 12 months, limiting the ability to assess long-term LDL-c reductions and clinical outcomes. Lastly, patients with PAD and those with cerebrovascular disease were underrepresented, potentially limiting the generalizability of our findings to the broader ASCVD population.

Future studies should prioritise digital health interventions primarily targeting lipid control and LDL-c reduction as primary outcomes. Larger, long-term trials are needed to identify which digital health characteristics are most effective for sustained engagement and lipid management. Identification of these factors is essential to optimize long-term impact of digital health, as larger LDL-c reductions may emerge over extended study periods. Remote monitoring remains underexplored in lipid management, unlike more established use in conditions such as heart failure,^47^ hypertension,^48^ diabetes,^49^^,^^50^ and arrhythmias.^51^ This approach could tackle common challenges in lipid management, such as suboptimal follow-up and treatment optimisation. Finally, evaluation of implementation factors, including feasibility, acceptability, adherence, scalability, and cost-effectiveness is crucial to guide large-scale adoption into clinical practice.

In conclusion, digital health interventions can be effective in reducing LDL-c, improving target attainment, and support therapy adherence among patients with ASCVD. Integrating digital health in clinical practice as part of CV risk management might therefore strengthen secondary prevention strategies. Multi-component interventions, incorporating elements of telemonitoring, high-frequency delivery, and bidirectional communication may be among the most effective for LDL-c reduction. Further long-term, high-quality pragmatic trials using digital health for lipid management are necessary to confirm these results and pave the way to implementation and improve long-term outcomes.

## Contributors

YS, AM, and MW conceptualised the study and designed the methodology. YS, DB, and MW performed the screening process and study selection. YS and DB performed data extraction and analysis. YS and MW drafted the manuscript. MS, GS, MW and FM contributed to the interpretation of the data and provided clinical input. MW contributed to funding acquisition. All authors contributed to critically revising the manuscript and had access to the data. YS, DB and MW verified the underlying data. All authors agreed to the final version of the manuscript and to the submission for publication.

## Data sharing statement

This systematic review and meta-analysis used extracted data from published studies and no individual patient data. All data analysed in this study are included in the article and its Supplementary appendix file.

## Declaration of interests

All authors declare no competing interests.
