# Supplementary material for: Digital health interventions for lipid management in atherosclerotic cardiovascular disease: a systematic review and meta-analysis of randomised controlled trials
Source: eClinicalMedicine. 2026 Apr 10;94:103886. doi: 10.1016/j.eclinm.2026.103886 (PMC13091938; doi:10.1016/j.eclinm.2026.103886)
Supplement: Appendix [file mmc1.docx]

**Appendix**

**Digital health interventions for lipid management in atherosclerotic cardiovascular disease: a systematic review and meta-analysis of randomized controlled trials**

Youri Schut, Danique G.B. Buhler, Marjolein Snaterse-Zuidam, Arjan Malekzadeh, G. Aernout Somsen, Fabrice M.A.C. Martens, Michiel M. Winter

**Table of contents**

Search strategy ……………………………………………………………………………….. 2

Eligibility criteria for study inclusion ………………………………………………………. 10

Statistical conversions ………………………………………………………………………. 11

List of excluded studies based on full-text screening with reasons for exclusion ………….. 12

Number of included studies per country and income economy …………………………….. 15

Risk of bias assessment ……………………………………………………………………... 16

LDL-c subgroup analysis by use of telemonitoring ………………………………………… 18

LDL-c subgroup analysis by type of communication ………………………………………. 19

LDL-c subgroup analysis by frequency …………………………………………………….. 20

LDL-c subgroup analysis by intervention duration ……………………………………...… 21

LDL-c subgroup analysis by income-economy …………………………………………….. 22

LDL-c subgroup analysis by baseline LDL-c levels ………………………………………... 23

Sensitivity analyses for LDL-c outcome ……………………………………………………. 24

Publication bias for LDL-c outcome ………………………………………………………... 25

**Search strategy**

| Database(s): Ovid MEDLINE(R) ALL 1946 to May 23, 2025 | |
| --- | --- |
| Search Strategy: |  |
| # | Searches |
| 1 | exp Atherosclerosis/ or exp Plaque, Atherosclerotic/ or exp Cardiovascular Diseases/ or exp Cardiac Rehabilitation/ or exp Coronary Disease/ or exp Myocardial Infarction/ or exp Acute Coronary Syndrome/ or exp Cerebrovascular Disorders/ or exp Stroke/ or exp Peripheral Arterial Disease/ or exp Myocardial Ischemia/ or *Heart Failure/ or exp Ischemic Attack, Transient/ |
| 2 | (atheromatos?s or atherosclero* or ASVD or ASCVD or Atherogenes?s).ti,ab,kf. |
| 3 | (Atheroma adj2 (formation or plaque*)).ti,ab,kf. |
| 4 | ((vascular or arterial) adj2 (plaque adj2 form*)).ti,ab,kf. |
| 5 | (intima adj2 plaque*).ti,ab,kf. |
| 6 | (CVA or cerebrovasculopathy or ((cerebrovascular or brain or cerebr* or intracranial*) adj3 (accident* or isch?emi* or apoplex* or attack* or stroke* or infarct* or lesion* or emboli* or insufficienc* or occlusion*))).ti,ab,kf. |
| 7 | (peripheral adj2 (artery or arterial) adj2 disease*).ti,ab,kf. |
| 8 | ((Acute adj2 Coronary adj2 Syndrome) or ACS).ti,ab,kf. |
| 9 | ((Coronary or heart or cadiovascular* or myocardial) adj2 (event* or disease* or stroke* or infarct* or isch?emi* or attack* or failure or rehab*)).ti,ab,kf. |
| 10 | (TIA or (attack adj3 "transient isch?emi*") or ((cerebral or brain or cerebral) adj2 isch?emi* adj2 transient)).ti,ab,kf. |
| 11 | or/1-10 |
| 12 | exp Telemedicine/ or exp telecommunications/ or remote consultation/ or exp Telephone/ or exp Mobile Applications/ or *Reminder Systems/ or exp user-computer interface/ or exp Computer Communication Networks/ or exp Telemetry/ or exp Monitoring, Ambulatory/ |
| 13 | ((tele or electronic or "e" or digital* or virtual* or online or remote or mobile) adj2 (medicine or referral* or cardiology or consult* or diagnos* or monitor* or pathology or pharmacy or rehab* or therapy or health or care or healthcare or medicine or nursing or communicat* or follow-up or followup)).ti,ab,kf. |
| 14 | (telemedicine or telecardiology or telemetr* or teleconsult* or telediagnos* or telemonitor* or telepathology or telepharmacy or telerehab* or telesurgery or teletherapy or telehealth or telecare or telehealthcare or telemedicine or telenursing or ehealth or "E-consult*" or telereferral* or telecommunicat* or mhealth or (video adj2 (consult* or communicat* or healthcare or health or care))).ti,ab,kf. |
| 15 | (telephone* or phone* or smartphone* or (smart adj1 phone*) or (text adj1 messag*) or SMS or (reminder adj1 system*)).ti,ab,kf. |
| 16 | ((computer or social media or virtual* or digital* or online) adj2 (communicat* or system* or assistan*)).ti,ab,kf. |
| 17 | ((Mobile or phone or smartphone*) adj2 (Application* or app* or software*)).ti,ab,kf. |
| 18 | (user adj2 computer adj2 interface).ti,ab,kf. |
| 19 | ((wearable or ambulatory or outpatient* or portable or home) adj2 monitor*).ti,ab,kf. |
| 20 | or/12-19 |
| 21 | exp Secondary Prevention/ |
| 22 | ((secondary or relaps* or recurren* or disease* or follow-up or followup or complication*) adj2 (prevent* or risk manag* or screen* or surveill*)).ti,ab,kf. |
| 23 | or/21-22 |
| 24 | exp Dyslipidemias/ or exp Lipoproteins/ or exp Hyperlipidemias/ or exp Hypolipoproteinemias/ or exp Cholesterol/ or exp Triglycerides/ or exp Patient Readmission/ or exp Medication Adherence/ or exp Mortality/ |
| 25 | (lipid* or dyslip?emia* or dyslipoproteinemia* or hyperlip?emia* or lip?emia* or triglyceride* or tryglyceride* or triacylglycerol* or hypolipoproteinemia* or hyperlipoproteinemia* or cholesterol* or epicholesterol or fatty acid* or lipoprotein* or LDL* or HDL*).ti,ab,kf. |
| 26 | (rehospitali* or readmission).ti,ab,kf. |
| 27 | ((medication* or drug* or therap* or treatment* or intervention* or dose or dosage) adj2 (compli* or persist* or concordance or adher*)).ti,ab,kf. |
| 28 | (death* or mortalit* or fatalit*).ti,ab,kf. |
| 29 | (MACE or ((cardiac or cardiovascular) adj2 (event* or outcome* or followup or "follow up" or incident*))).ti,ab,kf. |
| 30 | (adverse adj2 (event* or incident* or outcome*)).ti,ab,kf. |
| 31 | or/24-30 |
| 32 | 11 and 20 and 23 and 31 |

| Database(s): Embase Classic+Embase 1947 to 2025 May 23 | |
| --- | --- |
| Search Strategy: | |
| # | Searches |
| 1 | exp atherosclerosis/ or exp atherosclerotic plaque/ or exp cardiovascular disease/ or exp heart rehabilitation/ or exp coronary artery disease/ or exp heart infarction/ or exp acute coronary syndrome/ or exp cerebrovascular disease/ or exp cerebrovascular accident/ or exp peripheral arterial disease/ or exp heart muscle ischemia/ or *heart failure/ or exp transient ischemic attack/ |
| 2 | (atheromatos?s or atherosclero* or ASVD or ASCVD or Atherogenes?s).ti,ab,kf. |
| 3 | (Atheroma adj2 (formation or plaque*)).ti,ab,kf. |
| 4 | ((vascular or arterial) adj2 (plaque adj2 form*)).ti,ab,kf. |
| 5 | (intima adj2 plaque*).ti,ab,kf. |
| 6 | (CVA or cerebrovasculopathy or ((cerebrovascular or brain or cerebr* or intracranial*) adj3 (accident* or isch?emi* or apoplex* or attack* or stroke* or infarct* or lesion* or emboli* or insufficienc* or occlusion*))).ti,ab,kf. |
| 7 | (peripheral adj2 (artery or arterial) adj2 disease*).ti,ab,kf. |
| 8 | ((Acute adj2 Coronary adj2 Syndrome) or ACS).ti,ab,kf. |
| 9 | ((Coronary or heart or cadiovascular* or myocardial) adj2 (event* or disease* or stroke* or infarct* or isch?emi* or attack* or failure or rehab*)).ti,ab,kf. |
| 10 | (TIA or (attack adj3 "transient isch?emi*") or ((cerebral or brain or cerebral) adj2 isch?emi* adj2 transient)).ti,ab,kf. |
| 11 | or/1-10 |
| 12 | exp telemedicine/ or exp telehealth/ or exp telecommunication/ or exp video consultation/ or exp telephone/ or exp mobile application/ or *reminder system/ or exp computer interface/ or exp computer network/ or exp telemetry/ or exp ambulatory monitoring/ |
| 13 | ((tele or electronic or "e" or digital* or virtual* or online or remote or mobile) adj2 (medicine or referral* or cardiology or consult* or diagnos* or monitor* or pathology or pharmacy or rehab* or therapy or health or care or healthcare or medicine or nursing or communicat* or follow-up or followup)).ti,ab,kf. |
| 14 | (telemedicine or telecardiology or telemetr* or teleconsult* or telediagnos* or telemonitor* or telepathology or telepharmacy or telerehab* or telesurgery or teletherapy or telehealth or telecare or telehealthcare or telemedicine or telenursing or ehealth or "E-consult*" or telereferral* or telecommunicat* or mhealth or (video adj2 (consult* or communicat* or healthcare or health or care))).ti,ab,kf. |
| 15 | (telephone* or phone* or smartphone* or (smart adj1 phone*) or (text adj1 messag*) or SMS or (reminder adj1 system*)).ti,ab,kf. |
| 16 | ((computer or social media or virtual* or digital* or online) adj2 (communicat* or system* or assistan*)).ti,ab,kf. |
| 17 | ((Mobile or phone or smartphone*) adj2 (Application* or app* or software*)).ti,ab,kf. |
| 18 | (user adj2 computer adj2 interface).ti,ab,kf. |
| 19 | ((wearable or ambulatory or outpatient* or portable or home) adj2 monitor*).ti,ab,kf. |
| 20 | or/12-19 |
| 21 | exp Secondary Prevention/ |
| 22 | ((secondary or relaps* or recurren* or disease* or follow-up or followup or complication*) adj2 (prevent* or risk manag* or screen* or surveill*)).ti,ab,kf. |
| 23 | or/21-22 |
| 24 | exp dyslipidemia/ or exp "disorders of lipid and lipoprotein metabolism"/ or exp hyperlipidemia/ or exp hypolipemia/ or exp cholesterol/ or exp lipid blood level/ or exp triacylglycerol/ or exp low density lipoprotein/ or exp high density lipoprotein/ or exp lipid fingerprinting/ or exp hospital readmission/ or exp medication compliance/ or exp mortality/ |
| 25 | (lipid* or dyslip?emia* or dyslipoproteinemia* or hyperlip?emia* or lip?emia* or triglyceride* or tryglyceride* or triacylglycerol* or hypolipoproteinemia* or hyperlipoproteinemia* or cholesterol* or epicholesterol or fatty acid* or lipoprotein* or LDL* or HDL*).ti,ab,kf. |
| 26 | (rehospitali* or readmission).ti,ab,kf. |
| 27 | ((medication* or drug* or therap* or treatment* or intervention* or dose or dosage) adj2 (compli* or persist* or concordance or adher*)).ti,ab,kf. |
| 28 | (death* or mortalit* or fatalit*).ti,ab,kf. |
| 29 | (MACE or ((cardiac or cardiovascular) adj2 (event* or outcome* or followup or "follow up" or incident*))).ti,ab,kf. |
| 30 | (adverse adj2 (event* or incident* or outcome*)).ti,ab,kf. |
| 31 | or/24-30 |
| 32 | 11 and 20 and 23 and 31 |
| 33 | exp conference abstract/ or exp case report/ |
| 34 | 32 not 33 |

| Tue, may 27, 2025 Database - CINAHL Plus with Full Tekst | |
| --- | --- |
| # | Query |
| S28 | S11 and S19 and S20 and S27 |
| S27 | S21 or S22 or S23 or S24 or S25 or S26 |
| S26 | TI ( (adverse N2 (event* or incident* or outcome*)) ) OR AB ( (adverse N2 (event* or incident* or outcome*)) ) |
| S25 | TI ( (MACE or ((cardiac or cardiovascular) N2 (event* or outcome* or followup or "follow up" or incident*))) ) OR AB ( (MACE or ((cardiac or cardiovascular) N2 (event* or outcome* or followup or "follow up" or incident*))) ) |
| S24 | TI ( (death* or mortalit* or fatalit*) ) OR AB ( (death* or mortalit* or fatalit*) ) |
| S23 | TI ( ((medication* or drug* or therap* or treatment* or intervention* or dose or dosage) N2 (compli* or persist* or concordance or adher*)) ) OR AB ( ((medication* or drug* or therap* or treatment* or intervention* or dose or dosage) N2 (compli* or persist* or concordance or adher*)) ) |
| S22 | TI (( (lipid* or dyslip?emia* or dyslipoproteinemia* or hyperlip?emia* or lip?emia* or triglyceride* or tryglyceride* or triacylglycerol* or hypolipoproteinemia* or hyperlipoproteinemia* or cholesterol* or epicholesterol or fatty acid* or lipoprotein* or LDL* or HDL*) ) OR AB (( (lipid* or dyslip?emia* or dyslipoproteinemia* or hyperlip?emia* or lip?emia* or triglyceride* or tryglyceride* or triacylglycerol* or hypolipoproteinemia* or hyperlipoproteinemia* or cholesterol* or epicholesterol or fatty acid* or lipoprotein* or LDL* or HDL*) ) |
| S21 | (MH "Hyperlipidemia+") or MH "Lipoproteins+") or (MH "Hyperlipidemia+") or (MH "Cholesterol+") or (MM "Triglycerides") or (MM "Readmission") or (MM "Medication Compliance") or (MH "Mortality+") |
| S20 | TI ( ((secondary or relaps* or recurren* or disease* or follow-up or followup or complication*) N2 (prevent* or risk manag* or screen* or surveill*)) ) OR AB ( ((secondary or relaps* or recurren* or disease* or follow-up or followup or complication*) N2 (prevent* or risk manag* or screen* or surveill*)) ) |
| S19 | s12 or s13 or s14 or s15 or s16 or s17 or s18 |
| S18 | TI ( ((wearable or ambulatory or outpatient* or portable or home) N2 monitor*) ) OR AB ( ((wearable or ambulatory or outpatient* or portable or home) N2 monitor*) ) |
| S17 | TI (user N2 computer N2 interface) OR AB (user N2 computer N2 interface) |
| S16 | TI ( ((Mobile or phone or smartphone*) N2 (Application* or app* or software*)) ) OR AB ( ((Mobile or phone or smartphone*) N2 (Application* or app* or software*)) ) |
| S15 | TI ( ((computer or social media or virtual* or digital* or online) N2 (communicat* or system* or assistan*)) ) OR AB ( ((computer or social media or virtual* or digital* or online) N2 (communicat* or system* or assistan*)) ) |
| S14 | TI ( (telephone* or phone* or smartphone* or (smart N1 phone*) or (text N1 messag*) or SMS or (reminder N1 system*)) ) OR AB ( (telephone* or phone* or smartphone* or (smart N1 phone*) or (text N1 messag*) or SMS or (reminder N1 system*)) ) |
| S13 | TI ( ((tele or electronic or "e" or digital* or virtual* or online or remote or mobile) N2 (medicine or referral* or cardiology or consult* or diagnos* or monitor* or pathology or pharmacy or rehab* or therapy or health or care or healthcare or medicine or nursing or communicat* or follow-up or followup)) ) OR AB ( ((tele or electronic or "e" or digital* or virtual* or online or remote or mobile) N2 (medicine or referral* or cardiology or consult* or diagnos* or monitor* or pathology or pharmacy or rehab* or therapy or health or care or healthcare or medicine or nursing or communicat* or follow-up or followup)) ) |
| S12 | (MH "Telemedicine+") OR (MM "Telerehabilitation") OR (MH "Telehealth") or (MH "Telecommunications+") or (MM "Remote Consultation") or (MM "Telephone") or (MM "Mobile Applications") or (MM "Reminder Systems") or (MM "User-Computer Interface") or (MH "Computer Communication Networks+") or (MM "Telemetry") or (MH "Monitoring, Physiologic+") |
| S11 | s1 or s2 or s3 or s4 or s5 or s6 or s7 or s8 or s9 or s10 |
| S10 | TI ( (TIA or (attack N3 "transient isch?emi*") or ((cerebral or brain or cerebral) N2 isch?emi* N2 transient)) ) OR AB ( (TIA or (attack N3 "transient isch?emi*") or ((cerebral or brain or cerebral) N2 isch?emi* N2 transient)) ) |
| S9 | TI ( ((Coronary or heart or cadiovascular* or myocardial) N2 (event* or disease* or stroke* or infarct* or isch?emi* or attack* or failure or rehab*)) ) OR AB ( ((Coronary or heart or cadiovascular* or myocardial) N2 (event* or disease* or stroke* or infarct* or isch?emi* or attack* or failure or rehab*)) ) |
| S8 | TI ( ((Acute N2 Coronary N2 Syndrome) or ACS) ) OR AB ( ((Acute N2 Coronary N2 Syndrome) or ACS) ) |
| S7 | TI ( (peripheral N2 (artery or arterial) N2 disease*) ) OR AB ( (peripheral N2 (artery or arterial) N2 disease*) ) |
| S6 | TI ( (CVA or cerebrovasculopathy or ((cerebrovascular or brain or cerebr* or intracranial*) N3 (accident* or isch?emi* or apoplex* or attack* or stroke* or infarct* or emboli* or insufficienc* or occlusion*))) ) OR AB ( (CVA or cerebrovasculopathy or ((cerebrovascular or brain or cerebr* or intracranial*) N3 (accident* or isch?emi* or apoplex* or attack* or stroke* or infarct* or emboli* or insufficienc* or occlusion*))) ) |
| S5 | TI (intima N2 plaque*) OR AB (intima N2 plaque*) |
| S4 | TI ( ((vascular or arterial) N2 (plaque N2 form*)) ) OR AB ( ((vascular or arterial) N2 (plaque N2 form*)) ) |
| S3 | TI ( (Atheroma N2 (formation or plaque*)) ) OR AB ( (Atheroma N2 (formation or plaque*)) ) |
| S2 | TI ( (atheromatos?s or atherosclero* or ASVD or ASCVD or Atherogenes?s) ) OR AB ( (atheromatos?s or atherosclero* or ASVD or ASCVD or Atherogenes?s) ) |
| S1 | (MM "Atherosclerosis") or (MH "Cardiovascular Diseases+") or (MH "Rehabilitation, Cardiac+") or (MH "Coronary Disease+") or (MH "Myocardial Infarction+") or (MM "Acute Coronary Syndrome") or (MH "Cerebrovascular Disorders+") or (MH "Peripheral Vascular Diseases+") or (MH "Myocardial Ischemia+") or (MM "Heart Failure") or (MM "Cerebral Ischemia, Transient") |

|  | CENTRAL (Wiley), 27-5-2025 |
| --- | --- |
| ID | Search |
| #1 | MeSH descriptor: [Atherosclerosis] explode all trees |
| #2 | MeSH descriptor: [Plaque, Atherosclerotic] explode all trees |
| #3 | MeSH descriptor: [Cardiovascular Diseases] explode all trees |
| #4 | MeSH descriptor: [Cardiac Rehabilitation] explode all trees |
| #5 | MeSH descriptor: [Coronary Disease] explode all trees |
| #6 | MeSH descriptor: [Myocardial Infarction] explode all trees |
| #7 | MeSH descriptor: [Acute Coronary Syndrome] explode all trees |
| #8 | MeSH descriptor: [Cerebrovascular Disorders] explode all trees |
| #9 | MeSH descriptor: [Stroke] explode all trees |
| #10 | MeSH descriptor: [Peripheral Arterial Disease] explode all trees |
| #11 | MeSH descriptor: [Peripheral Arterial Disease] explode all trees |
| #12 | MeSH descriptor: [Heart Failure] this term only |
| #13 | MeSH descriptor: [Ischemic Attack, Transient] explode all trees |
| #14 | (atheromatos?s or atherosclero* or ASVD or ASCVD or Atherogenes?s):ti,ab,kw |
| #15 | (Atheroma NEAR/2 (formation OR plaque*)):ti,ab,kw |
| #16 | ((vascular OR arterial) NEAR/2 (plaque NEAR/2 form*)):ti,ab,kw |
| #17 | (intima NEAR/2 plaque*):ti,ab,kw |
| #18 | (CVA OR cerebrovasculopathy OR ((cerebrovascular OR brain OR cerebr* OR intracranial*) NEAR/3 (accident* OR isch?emi* OR apoplex* OR attack* OR stroke* OR infarct* OR lesion* OR emboli* OR insufficienc* OR occlusion*))):ti,ab,kw |
| #19 | (peripheral NEAR/2 (artery OR arterial) NEAR/2 disease*):ti,ab,kw |
| #20 | ((Acute NEAR/2 Coronary NEAR/2 Syndrome) OR ACS):ti,ab,kw |
| #21 | ((Coronary OR heart OR cardiovascular* OR myocardial) NEAR/2 (event* OR disease* OR stroke* OR infarct* OR isch?emi* OR attack* OR failure OR rehab*)):ti,ab,kw |
| #22 | (TIA OR (attack NEAR/3 "transient isch?emi*") OR ((cerebral OR brain OR cerebral) NEAR/2 isch?emi* NEAR/2 transient)):ti,ab,kw |
| #23 | {or #1-#22} |
| #24 | MeSH descriptor: [Telemedicine] explode all trees |
| #25 | MeSH descriptor: [Telecommunications] explode all trees |
| #26 | MeSH descriptor: [Remote Consultation] explode all trees |
| #27 | MeSH descriptor: [Telephone] explode all trees |
| #28 | MeSH descriptor: [Mobile Applications] explode all trees |
| #29 | MeSH descriptor: [Reminder Systems] this term only |
| #30 | MeSH descriptor: [User-Computer Interface] explode all trees |
| #31 | MeSH descriptor: [Computer Communication Networks] explode all trees |
| #32 | MeSH descriptor: [Telemetry] explode all trees |
| #33 | MeSH descriptor: [Monitoring, Ambulatory] explode all trees |
| #34 | ((tele OR electronic OR "e" OR digital* OR virtual* OR online OR remote OR mobile) NEAR/2 (medicine OR referral* OR cardiology OR consult* OR diagnos* OR monitor* OR pathology OR pharmacy OR rehab* OR therapy OR health OR care OR healthcare OR medicine OR nursing OR communicat* OR follow-up OR followup)):ti,ab,kw |
| #35 | (telemedicine OR telecardiology OR telemetr* OR teleconsult* OR telediagnos* OR telemonitor* OR telepathology OR telepharmacy OR telerehab* OR telesurgery OR teletherapy OR telehealth OR telecare OR telehealthcare OR telemedicine OR telenursing OR ehealth OR "E-consult*" OR telereferral* OR telecommunicat* OR mhealth OR (video NEAR/2 (consult* OR communicat* OR healthcare OR health OR care))):ti,ab,kw |
| #36 | (telephone* OR phone* OR smartphone* OR (smart NEAR/1 phone*) OR (text NEAR/1 messag*) OR SMS OR (reminder NEAR/1 system*)):ti,ab,kw |
| #37 | ((computer OR social media OR virtual* OR digital* OR online) NEAR/2 (communicat* OR system* OR assistan*)):ti,ab,kw |
| #38 | ((Mobile OR phone OR smartphone*) NEAR/2 (Application* OR app* OR software*)):ti,ab,kw |
| #39 | (user NEAR/2 computer NEAR/2 interface):ti,ab,kw |
| #40 | ((wearable OR ambulatory OR outpatient* OR portable OR home) NEAR/2 monitor*):ti,ab,kw |
| #41 | {or #24-#40} |
| #42 | MeSH descriptor: [Secondary Prevention] explode all trees |
| #43 | ((secondary or relaps* or recurren* or disease* or follow-up or followup or complication*) near/2 (prevent* or risk manag* or screen* or surveill*)):ti,ab,kw |
| #44 | {or #42-#43} |
| #45 | MeSH descriptor: [Dyslipidemias] explode all trees |
| #46 | MeSH descriptor: [Lipoproteins] explode all trees |
| #47 | MeSH descriptor: [Hyperlipidemias] explode all trees |
| #48 | MeSH descriptor: [Hyperlipidemias] explode all trees |
| #49 | MeSH descriptor: [Hyperlipidemias] explode all trees |
| #50 | MeSH descriptor: [Triglycerides] explode all trees |
| #51 | MeSH descriptor: [Patient Readmission] explode all trees |
| #52 | MeSH descriptor: [Medication Adherence] explode all trees |
| #53 | MeSH descriptor: [Mortality] explode all trees |
| #54 | (lipid* or dyslip?emia* or dyslipoproteinemia* or hyperlip?emia* or lip?emia* or triglyceride* or tryglyceride* or triacylglycerol* or hypolipoproteinemia* or hyperlipoproteinemia* or cholesterol* or epicholesterol or fatty acid* or lipoprotein* or LDL* or HDL*):ti,ab,kw |
| #55 | (rehospitali* or readmission):ti,ab,kw |
| #56 | ((medication* or drug* or therap* or treatment* or intervention* or dose or dosage) near/2 (compli* or persist* or concordance or adher*)):ti,ab,kw |
| #57 | (death* or mortalit* or fatalit*):ti,ab,kw |
| #58 | (MACE or ((cardiac or cardiovascular) near/2 (event* or outcome* or followup or "follow up" or incident*))):ti,ab,kw |
| #59 | (adverse near/2 (event* or incident* or outcome*)):ti,ab,kw |
| #60 | {or #45-#59} |
| #61 | #23 AND #41 AND #44 AND #60 in Trials |

**Eligibility criteria for study inclusion**

|  | **Inclusion criteria** | **Exclusion criteria** |
| --- | --- | --- |
| Population | Adults with ASCVD, defined as ACS, CVA/TIA, and PAD (secondary prevention) | Mixed patient populations without stratified results, patients without established ASCVD (primary prevention) |
| Intervention | Any type of digital health intervention including, but not restricted to, smartphone apps, text messaging, web platforms, and combinations | Digital health interventions that only consist of telephone calls/follow-up |
| Comparator | Standard care (face-to-face intervention, cardiac rehabilitation) | Standard care that also included components of digital health interventions |
| Outcomes | LDL-c levels | Not reporting LDL-c levels |
| Time | Intervention with a duration of at least 3 months | Intervention with a duration shorter than 3 months |
| Study design | Randomized controlled trials, observational studies | Systematic reviews, meta-analyses, retrospective studies, case-control, cross-sectional |
| Language | English | Non-English manuscripts |
| Published date | From inception to 27/5/2025 |  |
| Publication type | Manuscripts reporting findings of eligible study designs | Congress abstracts, protocols (rationale and design), animal studies, letters to editor |

**Statistical conversions**

Means and standard deviations (SD) were estimated through methods recommended by Wan et al. when studies reported outcomes as median with interquartile ranges. If standard error (SE) was reported instead of SD, this was converted to SD using SD = SE x √n. If 95% CI was reported instead of SD or SE, the SD was then calculated as described in chapter 7.7.3.2 of the Cochrane Handbook (Higgins et al., ). If lipid values were reported as mmol/L, they were multiplied by 38.66976 to convert them to mg/dL (for total cholesterol, HDL-c, and LDL-c) and by 88.57396 for triglycerides. For consistency across studies, a cholesterol conversion calculator was used <https://www.mdapp.co/cholesterol-conversion-calculator-600/>.

Higgins et al. (2011). Cochrane Handbook for Systematic Reviews of Interventions Version 5.1.0.

Wan et al. (2014). Estimating the sample mean and standard deviation from the sample size, median, range and/or interquartile range. BMC Med Res Methodol Vol. 14 Pages 135.

**List of excluded studies with reasons for exclusion based on full-text screening**

Protocol/conference abstract

Alex D et al (2014). Free communications 6: Preventive strategies, public awareness quality improvement secondary prevention of stroke. implementation of a point-of-care, cluster randomized trial using electronic health records (ecrt study). International Journal of Stroke.

Babu V et al (2023). LBO026 / #1536 MEDICATION ADHERENCE AND RISK FACTOR CONTROL FOR SECONDARY PREVENTION OF STROKE USING SMARTPHONE-BASED APPLICATION: A RANDOMIZED CONTROLLED TRIAL IN KERALA, SOUTH INDIA. International Journal of Stroke.

Claes J et al (2019). (#) PATHway-I: feasibility and preliminary efficacy of a technology-enabled home-based cardiac rehabilitation system. Acta Cardiologica.

Dorje T et al (2018). Smartphone and Social Media-based Cardiac Rehabilitation and Secondary Prevention (SMART-CR/SP) In China: results From A Randomised, Controlled Trial. Global Heart.

Kong X et al (2022). Remote telemedicine strategy based on multi-risks intervention by intelligent wearable health devices in elderly comorbidities patients with coronary heart disease. European Heart Journal, Volume 43, Issue Supplement_2, October 2022, ehac544.2813.

Lozano Martinez-Luengas I et al (2024). Utility of telephone text messages in secondary prevention after acute coronary syndrome. European Heart Journal - Volume 45, Issue Suppl 1.

Passaglia et al (2020). IMPACT OF TEXT MESSAGES IN A MIDDLE-INCOME COUNTRY TO PROMOTE SECONDARY PREVENTION AFTER ACUTE CORONARY SYNDROME (IMPACS): a RANDOMIZED TRIAL. JACC. Volume 75 Issue 11 Suppl 1.

Redfern J et al (2019). P5307 A consumer-direct digital health intervention for cardiovascular risk management in primary care: the Consumer Navigation of Electronic Cardiovascular Tools (CONNECT) randomised controlled trial. European Heart Journal. Volume 40, Issue Suppl 1.

Widmer R et al (2014). Digital health interventions improves cardiovascular risk factors and reduces rehospitalizations after usual cardiac rehabilitation. Circulation. Conference: American Heart Association's - Volume 130, Issue 0, pp.

Widmer R et al (2014). The augmentation of usual cardiac rehabilitation with an online and smartphone-based program improves cardiovascular risk factors and reduces rehospitalizations. Journal of the American College of Cardiology - Volume 1, Issue 0, pp. A1296.

Wienbergen H et al (2019). P6214 How to improve long-term prevention in young patients after myocardial infarction - The IPP-Y study. European Heart Journal - Volume 40, Issue 0, pp. 3825.

Zheng X et al (2019). Effect of cardiovascular health and text messaging (CHAT) on risk factor management in patients with coronary heart disease: a randomized clinical trial. Circulation: cardiovascular quality and outcomes. - Volume 12, Issue Suppl 1.

Ineligible study design

Ghavami M et al (2024). Effectiveness of the Green Heart Smartphone Application as a Self-Management Intervention for Hypertension and Dyslipidemia: A Randomized Clinical Trial. Archives of Iranian medicine - Volume 27(6), pp. 313-322.

Liu Z et al (2024). Effects of a Planned Web-Based Educational Intervention Based on the Health Belief Model for Patients With Ischemic Stroke in Promoting Secondary Prevention During the COVID-19 Lockdown in China: Quasi-Experimental Study. JMIR MHealth and UHealth - Volume 12.

Mathews T et al. (2017). Investigation of motivational interviewing and prevention consults to achieve cardiovascular targets (IMPACT) trial attainment of national lipid association guideline (NLA) Goals. Journal of Clinical Lipidology - Volume 11.

Michalski D et al. (2025). First experience in employing a complex digital support system accompanied by personal assistance to improve aftercare in patients with stroke or transient ischemic attack - results of the PostStroke-Manager feasibility study. Scientific reports - Volume 15.

Torri A et al. (2018). Promotion of Home-Based Exercise Training as Secondary Prevention of Coronary Heart Disease: A PILOT WEB-BASED INTERVENTION. Journal of Cardiopulmonary Rehabilitation & Prevention - Volume 38, Issue 4, pp. 253-258.

Non-English literature

Usova EI et al. (2022). An integrated approach for very high cardiovascular risk patients. Intermediate results [Russian]. Russian Journal of Cardiology - Volume 27, Issue 0, pp. 9-21.

Ineligible intervention

Garcia RV et al. (2022). Impact of a virtual lipid clinic on lipid-lowering therapy, LDL cholesterol levels, and outcomes in patients with acute coronary syndrome. Journal of Clinical Lipidology - Volume 16, Issue 0, pp. 635-642.

Xin Hu RN et al. (2014). Intensive Nursing Care by an Electronic Followup System to Promote Secondary Prevention After Percutaneous Coronary Intervention: A RANDOMIZED TRIAL. Journal of Cardiopulmonary Rehabilitation & Prevention - Volume 34, Issue 6, pp. 396-405.

Lester WT et al. (2006). Randomized controlled trial of an informatics-based intervention to increase statin prescription for secondary prevention of coronary disease. Journal of General Internal Medicine - Volume 21, Issue 1, pp. 22-29.

Nordmann A et al. (2001). A case-management program of medium intensity does not improve cardiovascular risk factor control in coronary artery disease patients: the Heartcare I trial. The American journal of medicine - Volume 110, Issue 0, pp. 543-550.

Wienbergen H et al. (2019). Effects of an intensive long-term prevention programme after myocardial infarction - a randomized trial. European Journal of Preventive Cardiology - Volume 26, Issue 5, pp. 522-530.

Ineligible population

Cicolini G et al. (2014). Efficacy of a nurse-led email reminder program for cardiovascular prevention risk reduction in hypertensive patients: a randomized controlled trial. International Journal of Nursing Studies - Volume 51, Issue 6, pp. 833-843.

Redfern J et al. (2020). A digital health intervention for cardiovascular disease management in primary care (CONNECT) randomized controlled trial. NPJ Digital Medicine - Volume 3, Issue 1, pp. 117.

Spassova L et al. (2016). Randomised controlled trial to evaluate the efficacy and usability of a computerised phone-based lifestyle coaching system for primary and secondary prevention of stroke. BMC Neurology - Volume 16, Issue 0, pp. 22.

Wong EML et al. (2022). Effectiveness of a Nurse-Led Support Programme Using a Mobile Application versus Phone Advice on Patients at Risk of Coronary Heart Disease – A Pilot Randomized Controlled Trial. Risk Management and Healthcare Policy - Volume 15, Issue 0, pp. 597-610.

Ineligible outcomes

Blasco A et al. (2012). Evaluation of a telemedicine service for the secondary prevention of coronary artery disease. Journal of Cardiopulmonary Rehabilitation and Prevention - Volume 32, Issue 1, pp. 25-31.

Dregan A et al. (2014). Point-of-care cluster randomized trial in stroke secondary prevention using electronic health records. Stroke - Volume 45, Issue 7, pp. 2066-2071.

Lear SA et al. (2015). Improving access to cardiac rehabilitation using the internet: a randomized trial. Studies in Health Technology and Informatics - Volume 209, Issue 0, pp. 58-66.

Santo K et al. (2018). The effects of a lifestyle-focused text-messaging intervention on adherence to dietary guideline recommendations in patients with coronary heart disease: an analysis of the TEXT ME study. International Journal of Behavioral Nutrition & Physical Activity - Volume 15, Issue 1.

No full text

Dregan A et al. (2014). Secondary prevention of stroke. Implementation of a point-of-care, cluster randomised trial using electronic health records (eCRT Study). Cerebrovascular Diseases - Volume 37, Issue 0, pp. 190.

Janssen H et al. (2023). Evaluation of the health service delivered secondary prevention program: supporting Lifestyle and Activity Modification after TIA (SLAM-TIA). International Journal of Stroke - Volume 18, Issue 2, pp. 8.

Snoek JA et al. (2021). The sustained effects of extending cardiac rehabilitation with a six-month telemonitoring and telecoaching programme on fitness, quality of life, cardiovascular risk factors and care utilisation in CAD patients: the TeleCaRe study. Journal of Telemedicine and Telecare - Volume 27, Issue 8, pp. 473-483.

**Number of included studies per country and income economy (according to The World Bank classification)**

| **Country** | **Number of studies** | **Income economy** |
| --- | --- | --- |
| India | 2 | Lower-middle |
| South-Korea | 1 | High |
| Colombia | 1 | Upper-middle |
| Australia | 3 | High |
| Spain | 1 | High |
| China | 5 | Upper-middle |
| Poland | 1 | High |
| Brazil | 1 | Upper-middle |
| New Zealand | 1 | High |
| The Netherlands | 1 | High |
| Hong Kong | 1 | High |

Source: The World Bank. World Bank Country and Lending Groups – Country Classification. Available from https://datahelpdesk.worldbank.org/knowledgebase/articles/906519. Accessed on August 30, 2025.

**Risk of bias assessment**


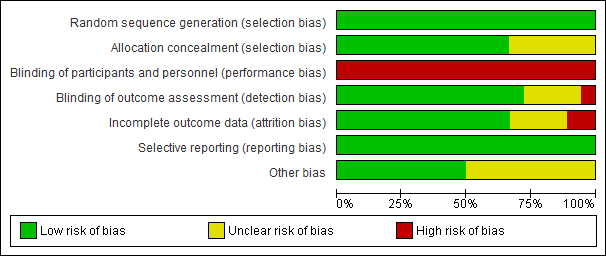


Risk of bias graph: judgements about each risk of bias item presented as percentages across all included studies.


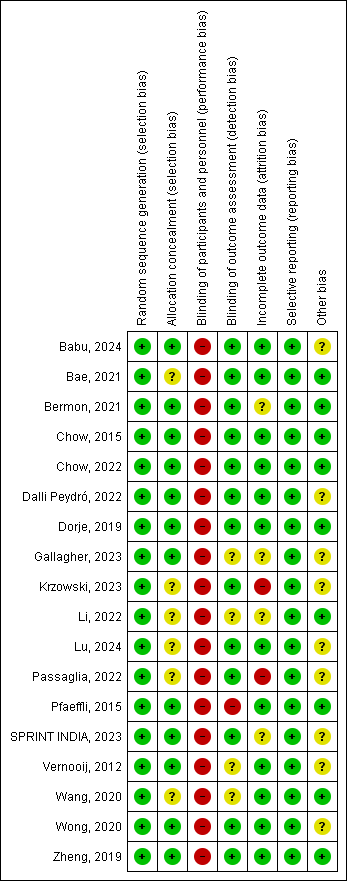


**LDL-c subgroup analysis by use of telemonitoring**

**
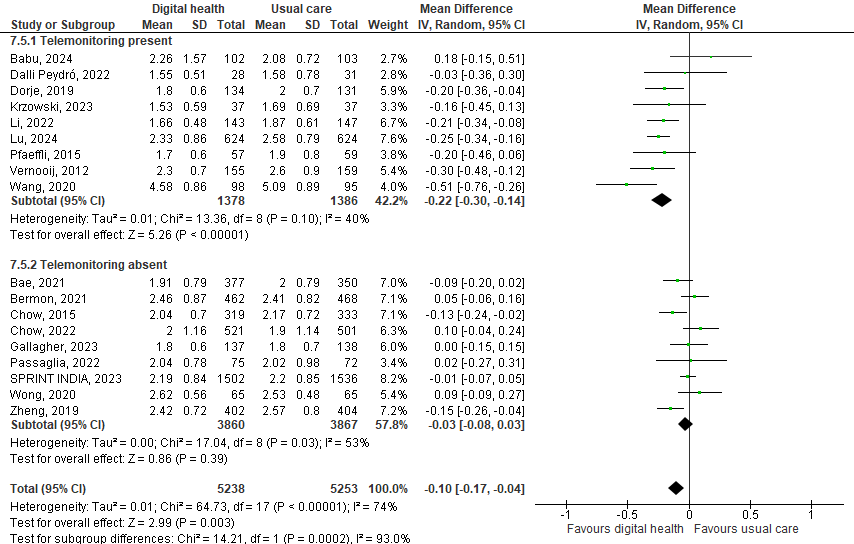
**

Forest plot of the mean difference in low-density lipoprotein cholesterol separated for integration of telemonitoring in the digital health intervention. Studies that used a form of telemonitoring in the intervention showed greater LDL-c reduction compared to studies that used interventions without telemonitoring. Subgroup differences were statistically significant (p<0.001).

**LDL-c subgroup analysis by type of communication of the intervention**


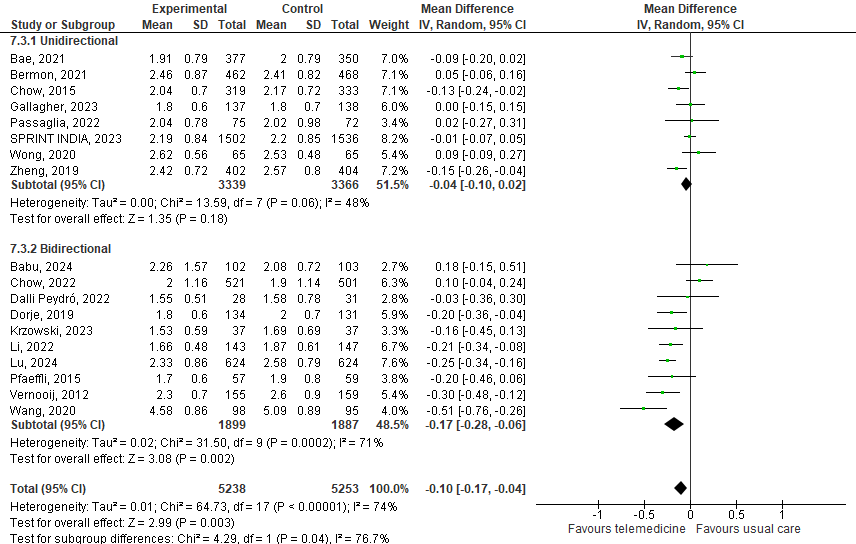


Forest plot of the mean difference in low-density lipoprotein cholesterol separated for type of communication of the intervention (unidirectional vs bidirectional). Bidirectional communication showed greatest LDL-c reduction potential compared to interventions that used unidirectional communication. Test for subgroup differences were statistically significant (p=0.04).

**LDL-c subgroup analysis by frequency of delivery of the intervention**

**
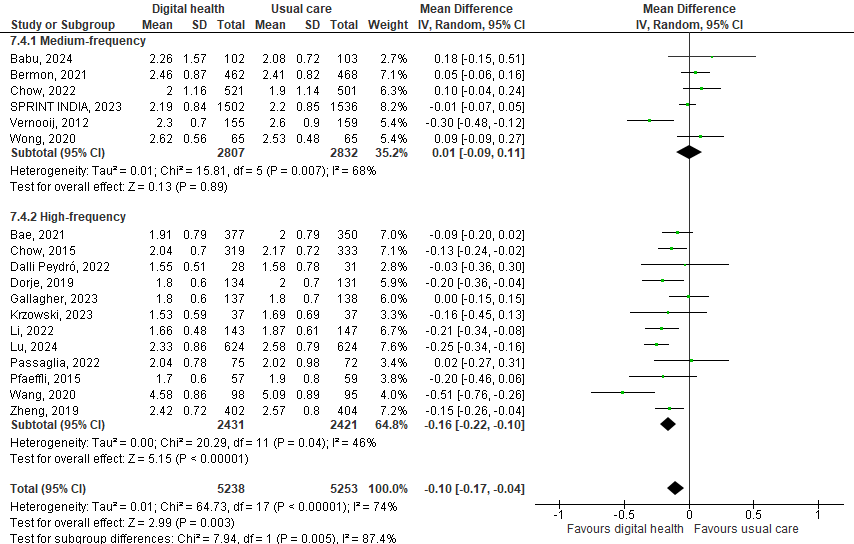
**

Forest plot of the mean difference in low-density lipoprotein cholesterol separated for frequency of delivery of the intervention (medium vs high-frequency). Studies that used interventions with high-frequency delivery showed greater potential to lower LDL-c compared to interventions that used medium-frequency delivery. The test for subgroup differences was statistically significant (p=0.005).

**LDL-c subgroup analysis by intervention duration**


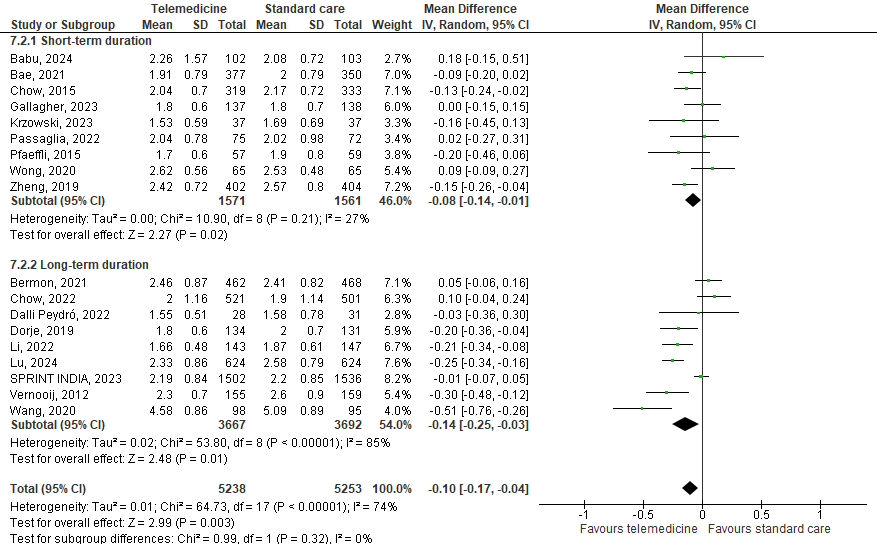


Forest plot for low-density lipoprotein cholesterol separated for short-term (≤6 months) and long-term (>6 months) intervention duration. Interventions with a duration longer than 6 months showed greater LDL-c reductions compared to interventions of 6 months and shorter. However, subgroup differences were not statistically significant (p=0.32).

**LDL-c subgroup analysis by income-economy**

**
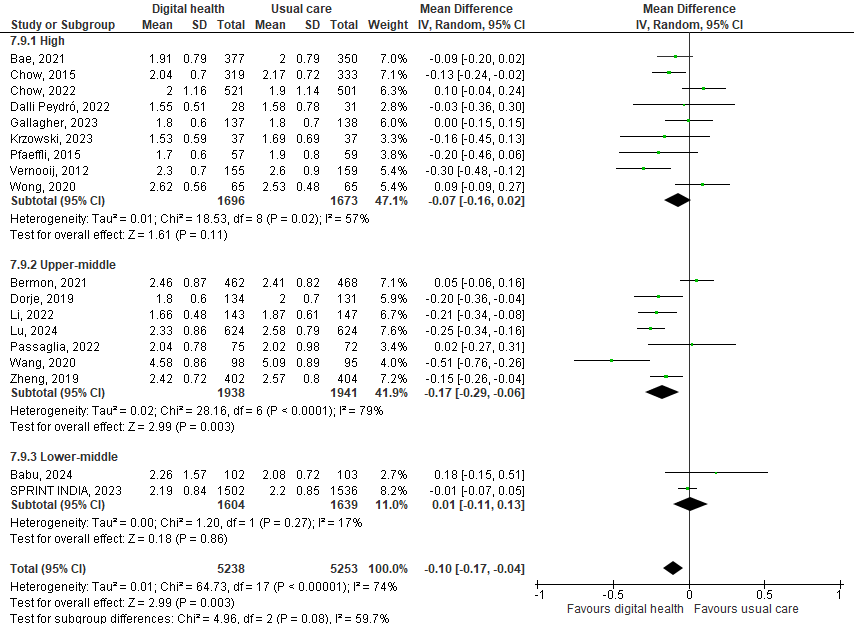
**

Forest plot for low-density lipoprotein cholesterol separated for high, upper-middle, and low-middle income countries**.** Studies performed in upper-middle income countries showed greatest LDL-c reduction compared to interventions in high- and lower-middle income countries. Test for subgroup differences was not statistically significant (p=0.08).

**LDL-c subgroup analysis by baseline LDL-c value**


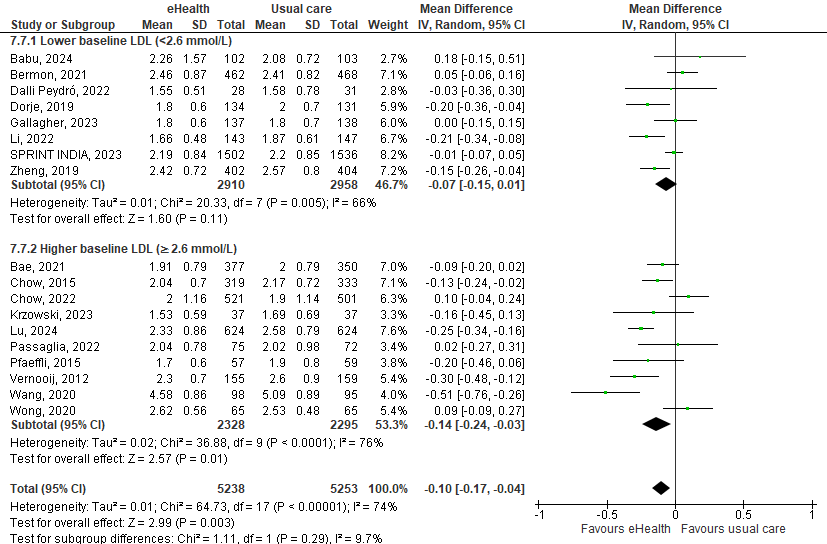


Forest plot for low-density lipoprotein cholesterol separated for the mean LDL-c values at baseline (lower, expressed as a mean LDL-c <2.6 mmol/L; higher, expressed as a mean LDL-c ≥2.6 mmol/L). Studies with participants with higher baseline LDL-c showed greater LDL-c reductions compared to studies with participants with lower LDL-c baseline values. Subgroup differences were not statistically significant (p=0.29).

**Sensitivity analyses for the LDL-c outcomes**

**
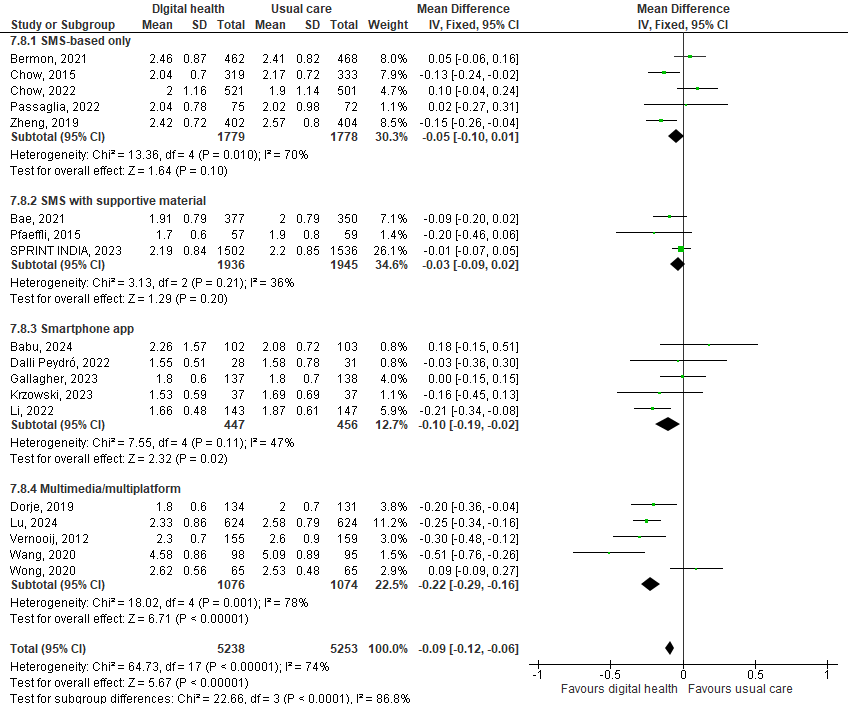
**

For the sensitivity analysis, a fixed-model was used instead of random-effects model. In this subgroup analyses, the fixed-effects model did not affect the direction of the associations, although it resulted in narrower confidence intervals for all groups and for the overall pooled estimate.

**Publication bias**

**
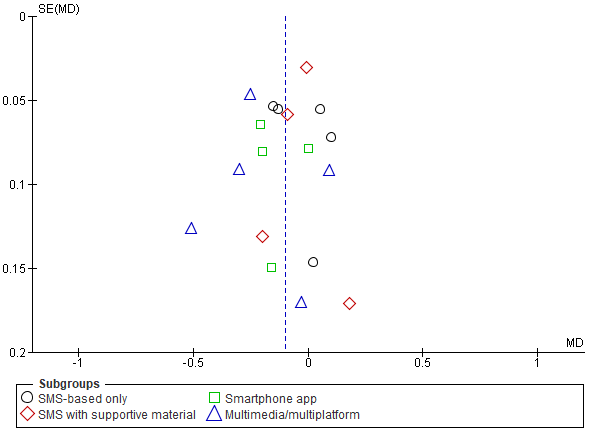
**

Funnel plot for risk of publication bias. Mean difference in LDL-c (expressed as mmol/L, x-axis) against the SE of the mean difference (y-axis). SE=standard error; MD=mean difference, SMS=short message service; LDL-c=low-density lipoprotein cholesterol
